# Examining the NEUROG2 lineage and associated gene expression in human cortical organoids

**DOI:** 10.1242/dev.202703

**Published:** 2025-01-16

**Authors:** Lakshmy Vasan, Vorapin Chinchalongporn, Fermisk Saleh, Dawn Zinyk, Cao Ke, Hamsini Suresh, Hussein Ghazale, Lauren Belfiore, Yacine Touahri, Ana-Maria Oproescu, Shruti Patel, Matthew Rozak, Yutaka Amemiya, Sisu Han, Alexandra Moffat, Sandra E. Black, JoAnne McLaurin, Jamie Near, Arun Seth, Maged Goubran, Orly Reiner, Jesse Gillis, Chao Wang, Satoshi Okawa, Carol Schuurmans

**Affiliations:** ^1^ Sunnybrook Research Institute, Biological Sciences Platform, Hurvitz Brain Sciences Program, 2075 Bayview Ave, Toronto, ON M4N 3M5, Canada; ^2^ Department of Laboratory Medicine and Pathobiology, Medical Sciences Building, 1 King's College Cir, University of Toronto, Toronto, ON M5S 1A8, Canada; ^3^ Department of Biochemistry, Medical Sciences Building, 1 King's College Cir, University of Toronto, Toronto, ON M5S 1A8, Canada; ^4^ Department of Immunology, Medical Sciences Building, 1 King's College Cir, University of Toronto, Toronto, ON M5S 1A8, Canada; ^5^ Department of Physiology, University of Toronto, Medical Sciences Building, 1 King's College Cir, Toronto, ON M5S 1A8, Canada; ^6^ Terrence Donnelly Centre for Cellular and Biomolecular Research, 160 College St, Toronto, ON M5S 3E1, Canada; ^7^ Department of Medical Biophysics, 101 College St Suite 15-701, Toronto General Hospital, University of Toronto, Toronto, ON M5G 1L7, Canada; ^8^ Sunnybrook Research Institute, Physical Sciences Platform, Hurvitz Brain Sciences Program, 2075 Bayview Ave, Toronto, ON M4N 3M5, Canada; ^9^ Dr. Sandra Black Centre for Brain Resilience & Recovery, LC Campbell Cognitive Neurology Unit, Sunnybrook Research Institute, Toronto, ON M4N 3M5, Canada; Hurvitz Brain Sciences Program; ^10^ Department of Medicine (Neurology) (SEB), University of Toronto, Toronto, ON M5S 3H2, Canada; ^11^ Departments of Molecular Genetics and Molecular Neuroscience, Weizmann Institute of Science, 76100 Rehovot, Israel; ^12^ Pittsburgh Heart, Lung, and Blood Vascular Medicine Institute, University of Pittsburgh School of Medicine, Pittsburgh, PA 15261, USA; ^13^ Department of Computational and Systems Biology, University of Pittsburgh School of Medicine, Pittsburgh, PA 15213, USA; ^14^ McGowan Institute for Regenerative Medicine, University of Pittsburgh School of Medicine, Pittsburgh, PA 15219, USA

**Keywords:** Cortical organoid, CRISPR gene editing, Lineage tracing, Proneural transcription factor, *NEUROG2*, Extracellular matrix, Human-specific features

## Abstract

Proneural genes are conserved drivers of neurogenesis across the animal kingdom. How their functions have adapted to guide human-specific neurodevelopmental features is poorly understood. Here, we mined transcriptomic data from human fetal cortices and generated from human embryonic stem cell-derived cortical organoids (COs) to show that *NEUROG1* and *NEUROG2* are most highly expressed in basal neural progenitor cells, with pseudotime trajectory analyses indicating that *NEUROG1*-derived lineages predominate early and *NEUROG2* lineages later. Using ChIP-qPCR, gene silencing and overexpression studies in COs, we show that *NEUROG2* is necessary and sufficient to directly transactivate known target genes (*NEUROD1*, *EOMES*, *RND2*). To identify new targets, we engineered *NEUROG2-mCherry* knock-in human embryonic stem cells for CO generation. The mCherry-high CO cell transcriptome is enriched in extracellular matrix-associated genes, and two genes associated with human-accelerated regions: *PPP1R17* and *FZD8*. We show that NEUROG2 binds *COL1A1*, *COL3A1* and *PPP1R17* regulatory elements, and induces their ectopic expression in COs, although *NEUROG2* is not required for this expression. *Neurog2* similarly induces *Col3a1* and *Ppp1r17* in murine P19 cells. These data are consistent with a conservation of *NEUROG2* function across mammalian species.

## Teaser

**Summary:** Analysis of human cortical organoids reveals that *NEUROG1* lineages prevail early and *NEUROG2* lineages later, and that *NEUROG2* targets include *COL* genes and *PPP1R17*, a human-accelerated region-associated gene.

## INTRODUCTION

Rodent models are used to study how the six-layered neocortex (‘cortex’) develops, but do not recapitulate several features of the human brain, which has an increased size and complexity (Dehay and Huttner, 2024). In mammals, the ventricular zone (VZ) is populated by primary neural progenitor cells (NPCs), termed apical radial glia (aRG), which initially undergo direct neurogenesis to form deep-layer neurons (Moffat and Schuurmans, 2024; Taverna et al., 2014). Later on, aRG give rise to intermediate progenitor cells (IPCs) that form a more basally located subventricular zone (SVZ) and undergo indirect neurogenesis to populate upper layers (Haubensak et al., 2004; Kowalczyk et al., 2009; Miyata et al., 2004; Noctor et al., 2004, 2008). In human and non-human primate cortices, the SVZ is enlarged and divided into an inner (i) SVZ and outer (o) SVZ (Fietz et al., 2010; Hansen et al., 2010; Reillo et al., 2011). In the oSVZ, a population of basal RG (bRG) has expanded, which correlates with increased brain size and is a primary driver of cortical evolution (Dehay et al., 2015; Fietz et al., 2010; Hansen et al., 2010; Lui et al., 2011; Reillo and Borrell, 2012). In gyrencephalic cortices, IPCs are transit-amplifying cells, dividing multiple times before differentiating to produce many more upper-layer neurons than in rodents (Fernandez and Borrell, 2023; Fietz et al., 2010). To accommodate more neurons, cortical folds have developed, which increase overall surface area (Amin and Borrell, 2020; Borrell, 2018; Moffat and Schuurmans, 2024).

In humans, these prominent phenotypic changes are associated with altered neurodevelopmental gene expression, driven in part by fast-evolving cis-regulatory regions, termed human accelerated regions (HARs), to which trans-acting transcription factors (TFs) bind (Boyd et al., 2015; Capra et al., 2013; Doan et al., 2016; Girskis et al., 2021; Kamm et al., 2013; Lambert et al., 2011; Pollard et al., 2006; Prabhakar et al., 2006; Vanderhaeghen and Polleux, 2023; Wei et al., 2019; Won et al., 2019). HAR mutations in individuals with neurodevelopmental disorders is suggestive of their importance during brain development (Doan et al., 2016). For example, HARE5 is a human-specific regulatory sequence that elevates *FZD8* expression to increase Wnt signaling and expand cortical NPCs (Boyd et al., 2015). *PPP1R17* is a HAR-regulated gene that encodes a negative regulatory subunit for protein phosphatases such as PP1 and PP2A (Endo et al., 2003; Hall et al., 1999), which control the G1-to-S phase transition (Moura and Conde, 2019). PPP1R17 is highly expressed in cortical NPCs in primates and not other mammalian cortices, and drives a lengthened cell cycle characteristic of human corticogenesis when misexpressed in mouse cells (Girskis et al., 2021).

Proneural genes encode basic helix-loop-helix TFs that are conserved drivers of neurogenesis (Bertrand et al., 2002; Oproescu et al., 2021). In rodent cortices, *Neurog2* is the main proneural gene, and is required and sufficient to specify a glutamatergic neuronal identity (Fode et al., 2000; Han et al., 2018; Mattar et al., 2008; Oproescu et al., 2021; Schuurmans et al., 2004). *Neurog1* plays a more minor role in tempering the pace of early murine cortical neurogenesis (Han et al., 2018). To control fate decisions by cortical NPCs, Neurog1 and Neurog2 directly transactivate target genes, and function as pioneer TFs that open chromatin to facilitate binding by other neurogenic and lineage-specifying TFs (Aydin et al., 2019; Noack et al., 2022; Pereira et al., 2024; Smith et al., 2016). Neurog2 re-organizes the chromatin landscape at several levels, driving DNA demethylation at Neurog2 motifs, and increasing chromatin looping and accessibility (Manelli et al., 2024 preprint; Noack et al., 2022; Pereira et al., 2024). In mice, *Neurog2* is initially expressed in aRG and drives the transition from aRG to IPC, and later is expressed in IPCs (Britz et al., 2006; Kovach et al., 2013; Miyata et al., 2004; Ochiai et al., 2009). In fluorescence-activated cell sorting (FACS)-enriched NPCs from human fetal cortices, *NEUROG2* and its target genes are expressed at the highest levels in bRG and to a lesser extent in aRG (Johnson et al., 2015). Overexpression of *NEUROG2* in the ferret cortex similarly promotes the basal translocation of aRG (Johnson et al., 2015). Here, we used human embryonic stem cell (hESC)-derived cortical organoids (COs) to compare *NEUROG1* and *NEUROG2* expression, and assess the function of *NEUROG2* during human cortical neurogenesis.

## RESULTS

### *NEUROG1* and *NEUROG2* are expressed during human cortical development

In mouse cortical development, *Neurog2* is expressed between embryonic day (E) 10.5 to E17.5, encompassing the neurogenic period, whereas *Neurog1* expression begins at E10.5, but declines by E14.5 (Han et al., 2018; Li et al., 2012; Moffat et al., 2023 preprint). To compare mRNA levels in individual cortical cell types, we performed a pseudo-bulk analysis of single-cell RNA-sequencing (scRNA-seq) data collected from E10.5-E17.5 mouse cortices (Di Bella et al., 2021). Aggregated transcript read counts for *Neurog1* and *Neurog2* were higher in IPCs than in all other cell types*,* followed by aRG and migrating neurons (Fig. S1A). Within IPCs, *Neurog1* and *Neurog2* were initially expressed at roughly equivalent levels at E10.5, but by E13.5 *Neurog2* expression predominated (Fig. S1A). *Neurog2* transcript counts superseded *Neurog1* read counts in aRG, in migrating, immature and glutamatergic neurons, and in non-neuronal cells at all stages (Fig. S1A).

**Fig. 1.**
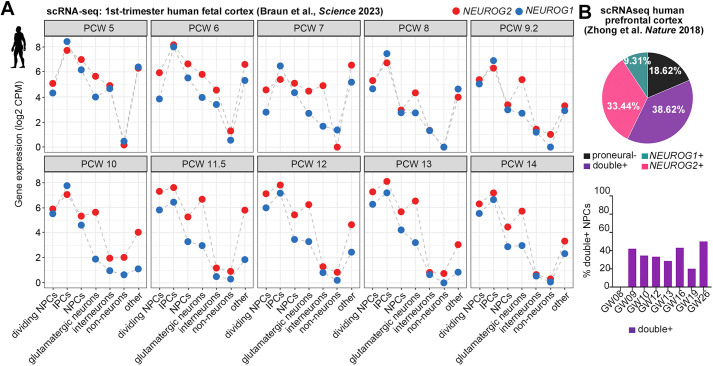
***NEUROG1* and *NEUROG2* expression in human fetal cortices.** (A) Pseudo-bulk analysis of *NEUROG1* and *NEUROG2* transcript counts in scRNA-seq data collected from post-conception weeks (PCW) 5-14 human cortices (Braun et al., 2023), showing log2 counts per million (CPM). (B) Distribution of *NEUROG1/NEUROG2* single and double-positive cells in scRNA-seq datasets from human fetal cortices between gestational week (GW) 8 and 26 (Zhong et al., 2018).

To assess *NEUROG2* and *NEUROG1* transcript levels during human cortical development, we performed a pseudo-bulk analysis of scRNA-seq data collected from post-conception weeks (PCW) 5-14 human cortices (Braun et al., 2023). *NEUROG2* and *NEUROG1* transcript counts were roughly equivalent and at the highest levels in IPCs at all stages (Fig. 1A). *NEUROG2* and *NEUROG1* transcripts were elevated in other NPC pools, including aRG and bRG with and without a proliferative gene signature (Fig. 1A). Finally, *NEUROG2* and to a lesser extent *NEUROG1* transcripts were detected at lower levels in glutamatergic neurons and ‘other’ cells (i.e. erythrocytes, vascular cells, placodes, fibroblasts), and in some neuroblasts, neurons and RG clusters that could not be merged with other annotated cell types (Fig. 1A).

To determine whether the same percentage of NPCs expressed *NEUROG1* and/or *NEUROG2*, we mined scRNA-seq data from gestational week (GW) 08 to GW26 human prefrontal cortices (Zhong et al., 2018). Of the cells assigned an NPC identity, the majority expressed *NEUROG2* (72.06%)*,* either together with *NEUROG1* (38.62%) or alone (33.44%) (Fig. 1B). In contrast, *NEUROG1* was expressed in fewer NPCs (47.93%), of which only 9.31% expressed *NEUROG1* alone (Fig. 1B)*.* Cortical NPCs co-expressing *NEUROG1* and *NEUROG2* persisted throughout the neurogenic window, from GW09 until GW26 (Fig. 1B). *NEUROG2* is therefore expressed in more human cortical NPCs than *NEUROG1* at the population level, even though relative transcript counts are similar within individual cells.

### *NEUROG1* and *NEUROG2* are primarily expressed in basal NPCs in cortical organoids

**Fig. 2.**
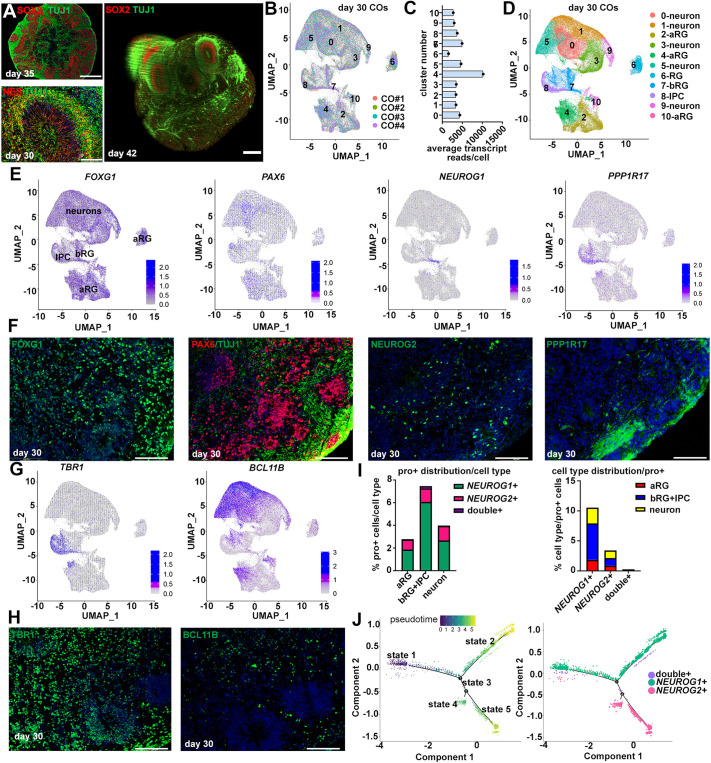
**Generation of COs and snRNA-seq analysis.** (A) Immunolabeling of day 35 and day 30 COs with SOX2 and TUJ1 or NES and TUJ1 (left), and 3D rendering of a tissue-cleared, day 42 CO immunolabeled with SOX2 and TUJ1 imaged with light sheet microscopy (right). (B) Overlay of uniform manifold approximation and projections (UMAPs) of snRNA-seq data collected from four independent sets of pooled day 30 COs. Numbers represent identified clusters. (C) Average transcript read counts per cell in each cluster. (D) UMAP showing the cluster distribution and manually annotated cluster identities. (E,F) Feature plots showing *FOXG1*, *PAX6*, *NEUROG1* and *PPP1R17* transcript distributions (E) and corresponding immunolabeling in day 30 COs (F). (G,H) Feature plots showing *TBR1* and *BCL11B* transcript distributions (G) and corresponding immunolabeling in day 30 COs (H). (I) Proportions of day 30 CO cell types expressing *NEUROG1* and/or *NEUROG2.* (J) Monocle3 lineage trajectory analysis of *NEUROG2*^+^, *NEUROG1*^+^ and double-positive cells in 30-day COs. aRG, apical radial glia; bRG, basal radial glia; IPC, intermediate progenitor cell. Scale bars: 400 mm (top left), 100 mm (bottom left), 200 mm (right) (A); 100 µm (F,H).

To assess proneural gene function in a human model system, we used directed differentiation to generate COs with a cortical identity from hESCs (Birey et al., 2017). Neural rosettes were observed in day 30-35 COs, comprising a central zone of SOX2^+^ and NES^+^ NPCs, and an external layer of TUJ1^+^ (β3-tubulin; TUBB3) neurons (Fig. 2A). To analyze CO structure, day 42 COs were optically cleared, co-immunostained with SOX2 and TUJ1, and imaged in wholemount using light sheet microscopy (Fig. 2A). A network of TUJ1^+^ axonal fibers encased the 42-day-old COs, and, in some instances, neural rosettes comprising an inner SOX2^+^ NPC layer and outer TUJ1^+^ neuronal layer appeared as external protrusions (Fig. 2A).

To confirm a cortical identity, we performed single-nuclear (sn)RNA-seq on four independent batches of five pooled COs per sample. A total of 103,459 cells passed quality control benchmarks, with an average of 4485±506 transcript reads per cell across the four groups (Fig. S2A,B). Batch correction was used to correct for technical variance, revealing that the four CO samples were highly correlated (Fig. 2B). Unbiased Seurat clustering stratified nuclei into ten cell clusters, all with high transcript read counts (Fig. 2C). Cluster identities were inferred based on cell type-specific markers and included aRG, bRG, IPCs and neurons (Fig. 2D, Table S1). Consistent with a forebrain identity, *FOXG1* was expressed in all cell clusters in day 30 COs (Fig. 2E,F). Four aRG clusters were identified (clusters 2, 4, 6, 10), of which clusters 4 and 10 expressed the highest levels of the pan-RG markers *PAX6*, *GLI3*, *SLC1A3*, *PROM1*, and *PARD3*, while clusters 2 and 4 expressed the highest levels of proliferation markers (*TOP2A*, *MKI67*, *CENPF*, *NUSAP1*) (Fig. 2E,F; Fig. S2C). Cluster 7 expressed the bRG marker *RASGRP1*, as well as *NEUROG1*, *NEUROG2* and known proneural target genes, such as *HES6*, *NHLH1* and *CBFA2T2* (Fig. 2E,F; Fig. S2C). A relatively small number of bRG were present in the day 30 COs in accordance with previous reports indicating that COs at 1-1.5 months of age primarily include aRG, with bRG only becoming predominant after 2 months (Uzquiano et al., 2022). Cluster 8 expressed *PPP1R17*, an IPC marker (Pollen et al., 2015), and markers of early-born neurons, including *TBR1*, *RELN* and *SLC17A6* (Fig. 2E-H; Fig. S2C). Finally, cells in clusters 0, 1, 3, 5 and 9 expressed neuronal markers, such as *BCL11B*, *ISL1* and *MEF2C* (Fig. 2G,H; Fig. S2C). Thus, *NEUROG1* and *NEUROG2* are most highly expressed in basal NPCs in day 30 COs, including bRG and IPCs, matching observations made in human fetal samples (Fig. 1).

### *NEUROG1* expression predominates early and *NEUROG2* later in a cortical organoid model of human cortical development

We used the snRNA-seq data from day 30 COs to interrogate proneural gene expression further. In day 30 COs, *NEUROG1* and/or *NEUROG2* were expressed in ∼8% of basal NPCs, including bRG and IPCs, in ∼3% of aRG, and in ∼4% of neurons (Fig. 2I). To understand lineage dynamics, we computationally isolated the 4896 cells expressing *NEUROG1* and/or *NEUROG2* and generated pseudotime trajectories (Fig. 2J; Fig. S3A). Pseudotime ordering of cells revealed a single main branch point and five cell states, with a relatively equal representation of cells from the four CO pools distributed within these states (Fig. 2J; Fig. S3B). State 1 and state 2 cells had the earliest pseudotime identities and predominantly included *NEUROG1* single-positive cells (Fig. 2J; Fig. S3C,D). State 3 was a small population of double-positive cells that appeared to be a transition step between early pseudotime states predominated by *NEUROG1* expression, and later pseudotime states (states 4 and 5) in which *NEUROG2* was instead expressed (Fig. 2J; Fig. S3C,D). Markers of aRG (*VIM*, *GLI3*, *PARD3*) were predominant in all cell states, whereas markers of bRG (*RASGRP1*, *EOMES*), proneural target genes (*HES6*) and markers of early-born layer 6 neurons (*FOXP2*) were highest in state 1 (Fig. S3E). In contrast, *BCL11B*, a layer 5 marker (Du et al., 2022), was expressed at higher levels in states 2, 4, and 5, with later pseudotime identities (Fig. S3E).

We reasoned that the unexpectedly higher expression levels of *NEUROG1* compared to *NEUROG2* in day 30 COs may be because this is a relatively early stage in CO development. To assess lineage relationships between *NEUROG1* and *NEUROG2* further, we generated pseudotime trajectories from mined scRNA-seq data collected from day 90 COs that were generated using an undirected Lancaster protocol (Sivitilli et al., 2020) (Fig. S4A). In these day 90 COs, *NEUROG2* expression predominated over *NEUROG1* (Fig. S4B,C). The resultant pseudotime ordering of cells along a lineage trajectory revealed a single branch point and three cell states (Fig. S4D,E). State 1 cells had the earliest pseudotime identity and included the highest fraction of *NEUROG1/NEUROG2* double-positive and *NEUROG1* single-positive cells, and the lowest fraction of *NEUROG2* single-positive cells (Fig. S4F,G). We determined that state 1 cells had the highest levels of aRG, bRG, and proliferating cell-associated transcripts (Fig. S4H). In intermediate state 2 cells, an increase in IPC marker expression coincided with an increase in the fraction of *NEUROG2* single-positive cells (Fig. S4H). Finally, state 3 cells, which had the latest pseudotime identities, predominantly expressed *NEUROG2* alone, with elevated levels of early neuronal marker transcripts, including deep-layer 5 and 6 markers (Fig. S4H). Thus, *NEUROG1* expression is confined to early stages of neural lineage development in human COs, and is enriched in aRG and bRG, whereas *NEUROG2-*expression increases later, as aRG/bRG mature into IPCs and neurons.

### *NEUROG2* is required and sufficient to transactivate neurogenic target genes in cortical organoids

**Fig. 3.**
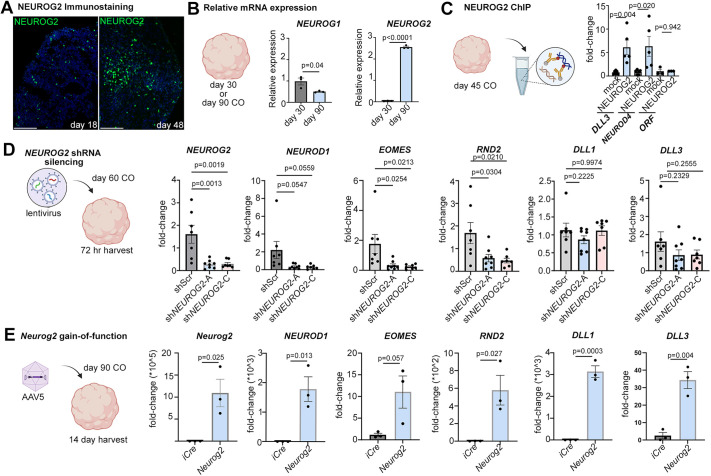
***NEUROG2* is necessary and sufficient to turn on neurogenic genes in COs.** (A) NEUROG2 immunolabeling of day 18 and day 48 COs. Scale bars: 100 µm. (B) qPCR of *NEUROG2* and *NEUROG1* in day 30 and day 90 COs. (C) NEUROG2 ChIP-qPCR (*n*=5), or mock control ChIP-qPCR (*n*=3), using day 45 COs, and qPCR amplified *DLL3* and *NEUROD4* promoter region binding sites in the eluted chromatin (*n*=5). An ORF amplified sequence was used as a negative control. (D) *NEUROG2* silencing in day 60 COs using lentiviral shRNA with a scrambled control sequence (shScr) and two shRNAs targeting *NEUROG2* (-A and -C) (*n*=7 each). COs were harvested after 72 h and the expression of *NEUROG2*, *NEUROD1*, *EOMES*, *RND2*, *DLL1* and *DLL3* was analyzed by qPCR. (E) *Neurog2* gain-of-function assay, using AAV5-GFAP-iCre (control) and AAV5-GFAP-*Neurog2*-iCre to transduce day 90 COs (*n*=3 each). COs were harvested after 14 days and the expression of *NEUROG2*, *NEUROD1*, *EOMES*, *RND2*, *DLL1* and *DLL3* was analyzed by qPCR. Graphs show mean±s.e.m. Unpaired Student's *t*-tests were used for pairwise comparisons. Significance was defined as *P*<0.05.

We focused on *NEUROG2* since its expression predominated in human fetal cortices and later-stage COs. We confirmed that NEUROG2 was expressed in COs, initially in a small number of cells at day 18, and in an increasing number of cells by day 48 (Fig. 3A). This increase was validated using qPCR, with almost a threefold increase in *NEUROG2* transcripts in day 90 versus day 30 COs (Fig. 3B). In contrast, *NEUROG1* transcript levels declined over this same period (Fig. 3B), consistent with the single-cell transcriptomic data. To assess *NEUROG2* function in COs, we used chromatin immunoprecipitation (ChIP) to examine whether NEUROG2 engages with known target genes (Fig. 3C). ChIP-qPCR performed on day 45 COs revealed that NEUROG2 binds to *DLL3* and *NEUROD4* promoter regions (Smith et al., 2016), whereas no binding was observed to an open reading frame (ORF) control sequence (Fig. 3C).

We next investigated whether *NEUROG2* was required to turn on known proneural target genes in human cortical cells using a shRNA-knockdown approach (Fig. 3D). Day 60 COs were transduced with lentiviral constructs carrying a shScrambled (shScr) control sequence and two shRNAs targeting endogenous *NEUROG2* (Fig. 3D). After 72 h post-transduction, we confirmed the silencing of *NEUROG2* with both shRNAs and demonstrated that *EOMES*, *NEUROD1* and *RND2*, known target genes, were also downregulated (Fig. 3D). In contrast, *DLL1* and *DLL3* transcript levels were not affected by *NEUROG2* silencing (Fig. 3D), likely due to their regulation by other TFs, such as the proneural TF encoded by *ASCL1* (Castro et al., 2006; Henke et al., 2009). Nevertheless, these data support the idea that *NEUROG2* has an essential role in driving neurogenic gene expression in COs, mimicking its requirement during murine cortical development (Fode et al., 2000; Schuurmans et al., 2004).

When overexpressed in E12.5 murine cortical NPCs, *Neurog2* is sufficient to induce the expression of downstream genes driving glutamatergic neuronal differentiation (Kovach et al., 2013). To examine whether *Neurog2* can similarly induce these target genes in hESC-derived COs, we transduced day 90 COs with adeno-associated virus (AAV) 5-GFAP promoter-containing vectors driving the expression of iCre as control or *Neurog2*-T2A-iCre (Fig. 3E). This expression vector drives gene expression in astrocytes and neural stem cells in the ventricular-subventricular zone of the adult mouse brain (Ghazale et al., 2022). COs were harvested after 14 days *in vitro*, and the ectopic expression of murine *Neurog2* was confirmed (Fig. 3E). Overexpression of *Neurog2* in day 90 COs was sufficient to induce the expression of known neurogenic target genes, including *NEUROD1*, *EOMES*, *RND2*, *DLL1*, and *DLL3* (Fig. 3E). Taken together, these data demonstrate that *NEUROG2* is necessary and sufficient to turn on neurogenic gene expression in hESC-derived COs, validating the use of this model system for further investigations of *NEUROG2* function.

### CRISPR engineering to generate *NEUROG2-mCherry-*KI hESCs for cortical organoid production

**Fig. 4.**
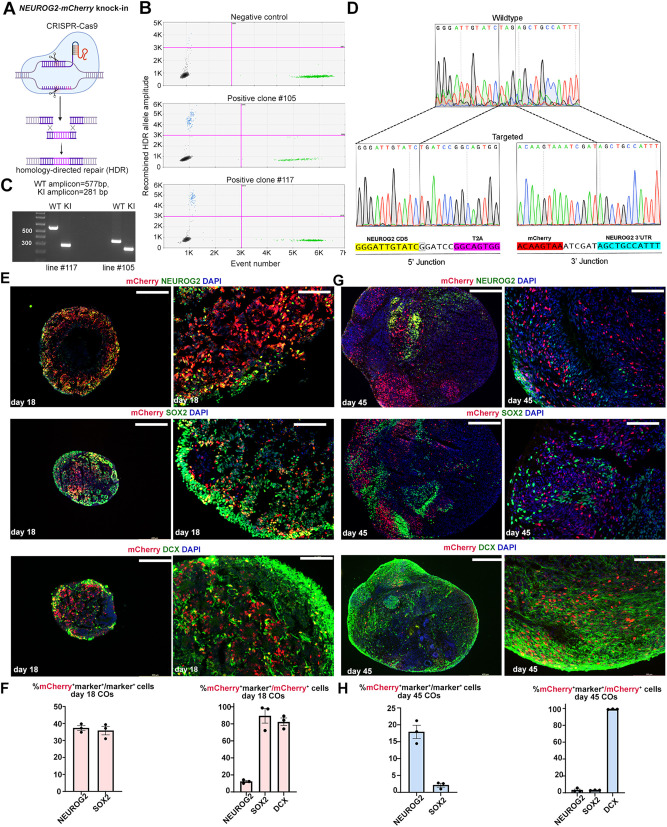
**Generation of *NEUROG2-mCherry* KI hESC-derived COs.** (A) CRISPR/Cas9-strategy to generate *NEUROG2-mCherry* KI hESCs by homology-directed repair (HDR). (B) ddPCR analysis of genomic DNA from sorted hESC-targeted cells. Raw droplet data of ddPCR measured for a negative control and two different positive clones indicating the increase in positive droplet count (blue dots) for the HDR sequence. (C) PCR genotyping of *NEUROG2* wild-type and *mCherry* KI alleles in line 117 and line 105, showing that both lines are heterozygous. Expected amplicon sizes were observed in line 117, but the two amplicons were smaller than expected in line 105, indicative of 3′ truncations. (D) Sanger sequencing of *NEUROG2-mCherry* KI targeted hESC clone 117 near the Cas9 target site. (E,F) Co-immunolabeling of day 18 COs with mCherry and NEUROG2, SOX2 or DCX (E), and associated quantification (*n*=3) (F). (G,H) Co-immunolabeling of day 45 COs with mCherry and NEUROG2, SOX2 or DCX (G), and associated quantification (*n*=3) (H). Graphs in F,H show mean±s.e.m. Scale bars: 400 µm (low-magnification images); 100 µm (high-magnification images).

To study the molecular phenotype of *NEUROG2*-expressing cells, we used clustered regularly interspaced short palindromic repeats (CRISPR)/Cas9 to engineer *NEUROG2-mCherry* knock-in (KI) reporter hESC lines (Fig. 4A). Individual hESC clones were screened for homology-directed repair (HDR) using Droplet Digital PCR, with 4/87 clones (4.6% efficiency) containing a *NEUROG2-mCherry* insertion (Fig. 4B). Lines 105 and 117 were expanded for further characterization, both of which lacked genomic abnormalities in the most commonly mutated regions (Fig. S5A), and expressed pluripotency genes (*OCT4*, *SOX2*, *NANOG*) at similar levels as the starting hESC population (Fig. S5B). Using PCR genotyping, line 117 was shown to be heterozygous with correct wild-type (577 bp) and mCherry-KI (281 bp) amplicon sizes (Fig. 4C). Sequencing of the 5′ and 3′ junctions of the Cas9 targeted site confirmed that no mutations were introduced into the *NEUROG2* locus at the junction sites in line 117 (Fig. 3D). However, while mCherry was inserted into the *NEUROG2* locus in line 105, smaller than expected amplicon sizes for both the wild-type and mCherry-KI allele were detected by PCR genotyping, indicative of truncations in the targeted 3′ untranslated region (UTR). Nevertheless, both line 105 and line 117 hESCs could generate COs using a directed differentiation protocol, forming neural rosettes comprising SOX2^+^ NPCs and TUJ1^+^ neurons at day 18 (Fig. S5D) and day 30 (Fig. S5E). Day 27-30 COs produced from the two modified *NEUROG2-mCherry* KI hESC lines had similar diameters as wild-type hESC-derived COs of the same age in culture (Fig. S5C).

To characterize the cellular composition of COs generated from *NEUROG2-mCherry* KI hESCs, we used the correctly targeted, heterozygous line 117. In COs cultured for 18 days *in vitro*, 37.3±1.4% of the NEUROG2^+^ cells co-expressed mCherry, whereas by day 45 only 17.9±1.9% of NEUROG2^+^ cells co-expressed the fluorescent reporter (Fig. 4E-H; Fig. S6A,B). The slow maturation kinetics of mCherry (∼52 min) may contribute to a delay in the onset of reporter expression (Guerra et al., 2022). Despite this delay, the persistence of mCherry expression has been exploited for short-term lineage tracing in the murine cortex using a *Neurog2-mCherry-*KI allele (Han et al., 2021), and in 2D neural cultures derived from *NEUROG2-TagRFP*-KI induced pluripotent stem cells (Park et al., 2022). We therefore characterized the co-expression of mCherry with SOX2, an NPC marker, and DCX, an immature neuronal marker, in *NEUROG2-mCherry* KI hESC-derived COs. In day 18 COs, 35.8±5.5% of SOX2^+^ NPCs co-expressed mCherry (Fig. 4E,F; Fig. S6A). In keeping with an NPC identity, the vast majority of mCherry^+^ cells co-expressed SOX2 (89.4±8.7%) and many had initiated DCX expression (82.5±4.4%) in day 18 COs (Fig. 4E,F; Fig. S6C,E). Notably, while DCX expression is restricted to newborn neurons in the developing murine cortex, DCX is also expressed in germinal zone NPCs during ferret cortical development (Wang et al., 2024). In contrast, by day 45, mCherry expression was only detected in 2.1±0.6% of SOX2^+^ NPCs, and within the overall mCherry^+^ population, only 3.1±0.2% of labeled cells were SOX2^+^ NPCs (Fig. 4G,H; Fig. S6D). Instead, 99.1±0.2% of mCherry^+^ cells co-expressed DCX at day 45, and since these cells are not SOX2 expressing, we infer that they are newborn neurons (Fig. 4G,H; Fig. S6F). The persistence of mCherry expression after *NEUROG2* expression declines allowed us to use mCherry to profile the phenotype of CO cells derived from *NEUROG2*^+^ NPCs.

### Transcriptomic analyses reveal a link between *NEUROG2* expression and the extracellular matrix in cortical organoids

**Fig. 5.**
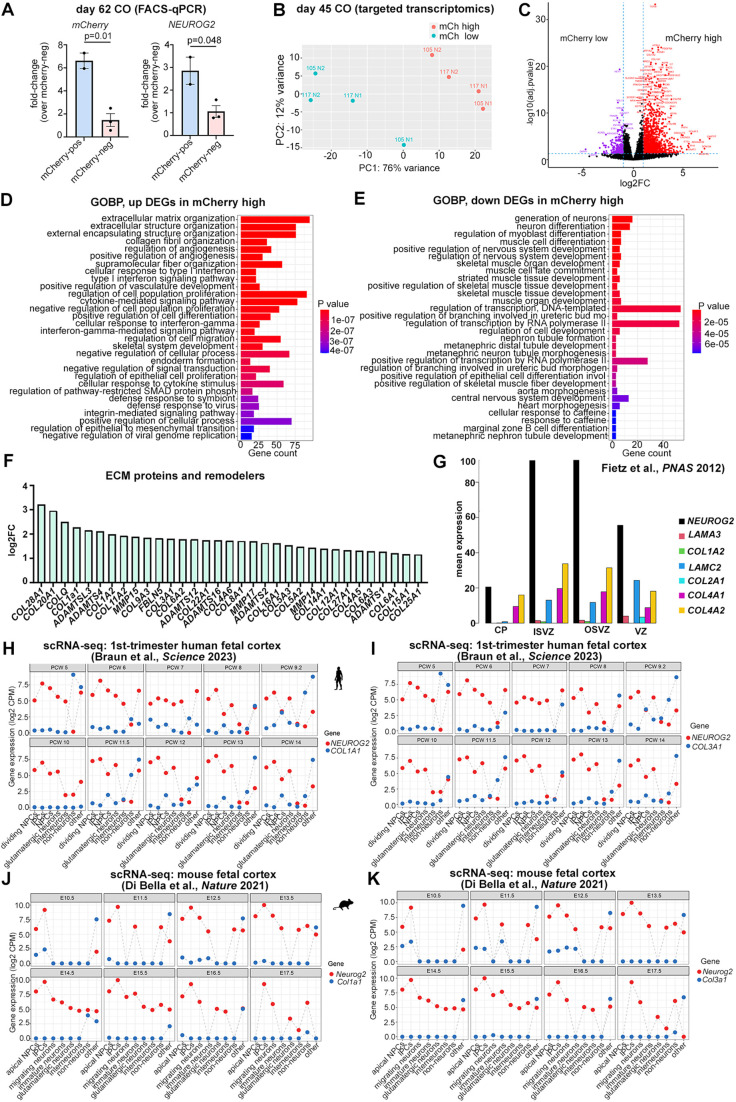
**Targeted transcriptomic analysis of *NEUROG2-mCherry* KI hESC-derived COs.** (A) qPCR to validate FACS-enrichment of *mCherry* and *NEUROG2* transcripts in mCherry-high versus mCherry-low cells (*n*=3 each). Data are mean±s.e.m. Unpaired Student's *t*-tests were used for pairwise comparisons. Significance was defined as *P*<0.05. (B) Principal component analysis of targeted transcriptomic data collected from two sets of day 45 COs generated from *NEUROG2-*mCherry KI hESC cell lines 105 and 117 (N1, N2), for a total of four replicate data sets. (C) Volcano plot showing enriched genes in mCherry-high versus mCherry-low day 45 CO cells. (D,E) Biological Process-Gene Ontology (GOBP) terms enriched in DEGs that were upregulated (D) or downregulated (E) in mCherry-high CO cells. (F) Bar graph showing log2FC values of DEGs encoding ECM proteins and remodelers. (G) Enrichment of *NEUROG2* and ECM gene transcripts in NPC compartments from microdissected human fetal cortical zones (Fietz et al., 2012). (H,I) Pseudo-bulk analysis of *NEUROG2* and *COL1A1* (H) and *NEUROG2* and *COL3A1* (I) transcript counts in scRNA-seq data collected from PCW 5-14 human cortices (Braun et al., 2023), showing log2CPM. (J,K) Pseudo-bulk analysis of *Neurog2* and *Col1a1* (J) and *Neurog2* and *Col3a1* (K) transcript counts in scRNA-seq data collected from E10.5 to E17.5 mouse cortices (Di Bella et al., 2021), showing log2CPM. BP, biological process; CO, cortical organoid; CP, cortical plate; DEGs, differentially expressed genes; ECM, extracellular matrix; GO, gene ontology; IPCs, intermediate progenitor cells; ISVZ, inner subventricular zone; mCh, mCherry; NPCs, neural progenitor cells; OSVZ, outer subventricular zone; VZ, ventricular zone.

To characterize gene expression in the *NEUROG2* lineage, we generated COs from *NEUROG2-mCherry* KI hESCs, using both lines 105 and 117 in two independent experiments. COs were harvested after 45-47 days *in vitro*, and mCherry-high and mCherry-low cells were FACS-enriched from pools of seven or eight COs. mCherry-high cells represented 3.5% of the total sorted cell pool in COs derived from hESC line 117, and 2.6% in COs from hESC line 105. The enrichment of *mCherry* and *NEUROG2* transcripts in mCherry-high versus -low cells was verified by Droplet Digital PCR (ddPCR) on RNA isolated from the sorted cells used for transcriptomics (Fig. S7A) and by qPCR on re-sorted day 62 COs for validation (Fig. 5A). We refer to sorted cells as mCherry-high and mCherry-low to acknowledge the low level of mCherry-expressing cells collected in the ‘negative’ sorted cells, and not to suggest that mCherry expression is at variable levels within each cell pool.

To profile gene expression, we used targeted transcriptome analysis covering 20,802 human genes, representing >95% of the UCSC reference genome. Principal component analysis of gene expression datasets revealed that mCherry-high versus mCherry-low cells were transcriptionally divergent and segregated from each other irrespective of the initiating hESC line or experimental day (Fig. 5B). We selectively assayed transcript counts for known genes involved in cortical development, focusing on transcripts with a log2 fold change (log2FC) >1 (i.e. a doubling in the original scaling) and with an adjusted *P*-value <0.05, corresponding to a false discovery rate cutoff of 0.05. A comparative analysis of differentially expressed genes (DEGs) in mCherry-high versus mCherry-low cells identified 1204 genes enriched in mCherry-high cells and 263 genes enriched in mCherry-low cells (Fig. 5C; Table S2). Gene ontology (GO) analysis of DEGs revealed an enrichment of biological process (BP) terms associated with the extracellular matrix (ECM) in mCherry-high cells, including ‘extracellular matrix organization’ and ‘collagen fibril organization’ (Fig. 5D; Table S3). Within the ECM-related GO-BP category: 0030198, 76/327 genes were among the DEGs enriched in mCherry-high cells. Included were collagens, and ADAM and matrix metalloproteases, which are involved in ECM remodeling (Fig. 5F).

### Assessing the transcriptional relationship between NEUROG2 and collagen genes

The enrichment of ECM-associated gene expression in the *NEUROG2-*mCherry lineage is also observed in human bRG (Pollen et al., 2015), with expansion of the ECM in the oSVZ providing a pro-proliferative niche for basal NPCs (Amin and Borrell, 2020; Arai et al., 2011; Fietz et al., 2012; Florio et al., 2015; Martinez-Martinez et al., 2016; Pollen et al., 2015). To validate an association between *NEUROG2* and ECM gene expression, we first mined a bulk RNA-seq dataset from the human fetal cortex (Fietz et al., 2012). In this dataset, several collagen genes, such as *COL1A2*, *COL4A1*, and *COL4A2*, were expressed at elevated levels in the VZ, iSVZ and oSVZ, in compartments in which *NEUROG2* transcript levels were also elevated (Fig. 5G). However, a pseudo-bulk analysis of PCW 5-14 human cortical scRNA-seq data (Braun et al., 2023) revealed that *COL1A1* and *COL3A1* expression was comparatively much lower than *NEUROG2* in aRG and bRG (Fig. 5H,I), a finding also observed in E10.5 to E17.5 mouse cortices (Di Bella et al., 2021) (Fig. 5J,K).

**Fig. 6.**
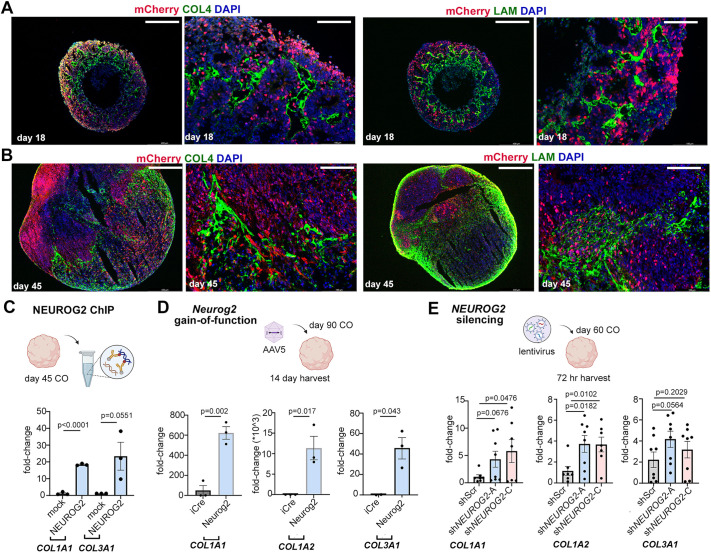
**Transcriptional relationship between NEUROG2 and *COL1A1*, *COL1A2* and *COL3A1*.** (A,B) Co-immunolabeling of day 18 (A) and day 45 (B) *NEUROG2*-*mCherry* KI hESC-derived COs with mCherry and the ECM markers collagen IV (COL4) or laminin (LAM). Scale bars: 400 µm (low-magnification images); 100 µm (high-magnification images). (C) NEUROG2 ChIP-qPCR (*n*=3), or mock control ChIP-qPCR (*n*=3), using day 45 COs. qPCR to quantify *COL1A1* and *COL3A1* promoter region binding sites and an ORF control sequence in the eluted chromatin. (D) *Neurog2* overexpression, using AAV5-GFAP-iCre (control) and AAV5-GFAP-Neurog2-iCre to transduce day 90 COs (*n*=3 each). COs were harvested after 14 days and the expression of *COL1A1*, *COL1A2* and *COL3A1* was analyzed by qPCR. (E) *NEUROG2* silencing in day 60 COs using lentiviral shRNA constructs, with a scrambled control sequence (shScr) or targeting *NEUROG2* (-A and -C) (*n*=7 each). COs were harvested after 72 h and the expression of *COL1A1*, *COL1A2* and *COL3A1* was analyzed by qPCR. Graphs show mean±s.e.m. Unpaired Student's *t*-tests were used for pairwise comparisons. Significance was defined as *P*<0.05.

To examine the expression of ECM proteins in the *NEUROG2* lineage, we co-immunostained *NEUROG2-mCherry* KI hESC-derived COs with mCherry and COL4 or laminin (LAM) antibodies. In day 18 COs, robust expression of COL4 and LAM was detected in circular formations in the center of the organoid, as well as in protrusions into the organoid periphery, where mCherry^+^ cells were concentrated (Fig. 6A). In higher magnification images, mCherry^+^ cells were surrounded by COL4^+^ and LAM^+^ protrusions, but there was limited overlap in expression (Fig. 6A). Similarly, in day 45 COs (Fig. 6B) and in optically cleared day 119 COs (Fig. S7B,C), COL4^+^ and LAM^+^ fibrils invaded the patches of mCherry-expressing cells, without obvious overlap in expression. There were also several regions with abundant mCherry^+^ cells that were devoid of ECM expression.

Given the lack of significant overlap between mCherry and ECM proteins, we set out to decipher the transcriptional relationship between NEUROG2 and ECM-related genes. By performing ChIP-qPCR on day 45 COs, we found that NEUROG2 bound upstream promoter regions for *COL1A1* and *COL3A1* (Qing et al., 2022) (Fig. 6C). To assess whether NEUROG2 was sufficient to induce the expression of ECM-related genes, we transduced day 90 COs with AAV5-GFAP-iCre (control) and AAV5-GFAP-*Neurog2*-T2A-iCre. After 14 days *in vitro*, *COL1A1*, *COL1A2* and *COL3A1* expression increased in response to *Neurog2* overexpression (Fig. 6D).

Finally, to determine whether *NEUROG2* was required to turn on collagen genes in human cortical cells, day 60 COs were transduced with lentiviral constructs carrying an shScr control sequence and two shRNAs targeting endogenous *NEUROG2* (Fig. 6E). After 72 h, we unexpectedly observed an increase in *COL1A1*, *COL1A2* and *COL3A1* transcripts with at least one *NEUROG2-*shRNA (Fig. 6E). *NEUROG2* is thus required to suppress collagen gene expression, with the caveat that the lentiviral silencing vector targets dividing NPCs and post-mitotic cells, and collagen genes may be differentially regulated in both cell types. Thus, the observed correlation between collagen gene expression and mCherry-high CO cells may reflect lineage maturation, with ECM genes upregulated as *NEUROG2* expression declines. However, since *NEUROG2* can also induce ectopic collagen gene expression, the relationship between *NEUROG2* and collagen gene expression is complex.

### Transcriptomic comparisons of mCherry-high and -low cells identify *PPP1R17* as a potential NEUROG2 target gene

We performed GO analysis of downregulated DEGs in mCherry-high cells and observed an over-representation of BP terms such as ‘generation of neurons’ and ‘neuron differentiation’ (Fig. 5E; Table S4). To understand why neurogenesis-related terms were downregulated in the mCherry-lineage, we performed a biased analysis of select neurogenic genes (Fig. S8). Most aRG, bRG, proliferating cell and pan-neuronal markers were expressed at roughly equivalent levels in mCherry-high and mCherry-low CO cells (Fig. S8A-E). Of the few genes enriched in mCherry-high cells, several are associated with the glutamatergic neuronal lineage, including *GAP43*, *SLC17A6* (*VGLUT2*), *SLC17A7* (*VGLUT1*), *RELN* and *PCP4* (Fig. S8E,F,H). In contrast, GABAergic neuronal lineage markers were enriched in mCherry-low cells, such as *ASCL1* and *GAD2*, as well as *BCL11B* and *FOXP2*, markers of GABAergic interneurons and deep-layer cortical neurons (Fig. S8G,H). Thus, a main difference between mCherry-high and mCherry-low cells is an association with glutamatergic or GABAergic neuronal lineages, respectively. These findings are consistent with the known role of *Neurog2* in specifying a glutamatergic neuronal fate in the cortex, and its repression of *Ascl1*, a GABAergic determinant (Fode et al., 2000; Kovach et al., 2013; Schuurmans et al., 2004).

**Fig. 7.**
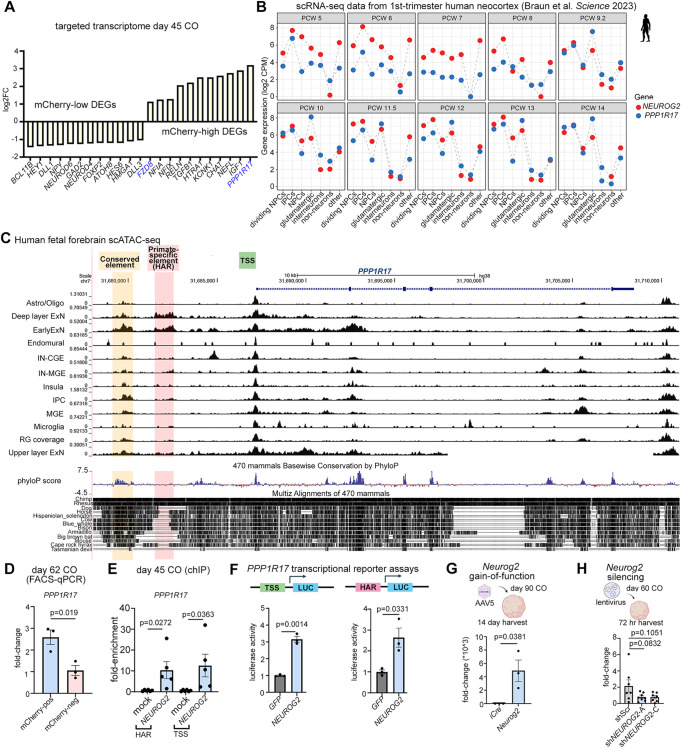
**NEUROG2 engages with *PPP1R17*-regulatory elements and is sufficient to induce *PPP1R17* transcription.** (A) Bar graph showing log2FC values of DEGs involved in neurogenesis in mCherry-high and in mCherry-low CO cells. (B) Pseudo-bulk analysis of *NEUROG2* and *PPP1R17* transcript counts in scRNA-seq data collected from PCW 5-14 human cortices (Braun et al., 2023), showing log2CPM. (C) Single-cell ATAC-seq profiling of the *PPP1R17* locus, showing accessible chromatin in regions and cell types in the developing human brain. Conserved accessible chromatin regions were identified in an upstream enhancer (yellow box) and surrounding the TSS (green box). A primate-specific HAR (red box) is mainly accessible in glutamatergic cortical lineages. A phyloP score was derived from multiple mammalian species, with negative scores indicative of accelerated evolution for the *PPP1R17*-HAR element in chimps and rhesus monkeys. (D) qPCR to validate FACS-enrichment of *PPP1R17* transcripts in mCherry-high versus mCherry-low cells (*n*=3 each). (E) NEUROG2 ChIP-qPCR (*n*=3), or mock control ChIP-qPCR (*n*=3), using day 45 COs. qPCR was used to quantify *PPP1R17*-HAR and -TSS binding sites in the eluted chromatin. (F) Transcriptional reporter assay in SHSY-5Y human neuroblastoma cells using pCIG2-*Neurog2* or pCIG2-GFP (negative control) expression vectors and luciferase (LUC) constructs with a minimal promoter carrying the *PPP1R17-*HAR or -TSS elements. (G) *Neurog2* gain-of-function assay, using AAV5-GFAP-*iCre* (control) and AAV5-GFAP-*Neurog2-iCre* to transduce day 90 COs (*n*=3 each). COs were harvested after 14 days and the expression of *PPP1R17* was analyzed by qPCR. (H) *NEUROG2* silencing in day 60 COs using lentiviral shRNA constructs, with a scrambled control sequence (shScr) or targeting *NEUROG2* (-A and -C) (*n*=7 each). COs were harvested after 72 h and *PPP1R17* expression was analyzed by qPCR. Graphs show mean±s.e.m. Unpaired Student's *t*-tests were used for pairwise comparisons. Significance was defined as *P*<0.05.

We examined the top DEGs in the mCherry-high and mCherry-low lineages more closely (Fig. 7A). *FZD8* (Boyd et al., 2015) and *PPP1R17* (Girskis et al., 2021) were of interest as they are controlled by known HARs (Fig. 7A). An enrichment of *PPP1R17* transcripts in the *NEUROG2* lineage was consistent with both of these genes being expressed in basal NPCs during human cortical development (this study; Girskis et al., 2021; Johnson et al., 2015). To compare *NEUROG2* and *PPP1R17* expression profiles further, we performed a pseudo-bulk comparison of scRNA-seq data collected from PCW 5-14 human cortices (Braun et al., 2023). *NEUROG2* and *PPP1R17* were expressed at roughly equivalent levels in all NPC pools, including IPCs, as well as in glutamatergic neurons and ‘other’ cells, especially after PCW 9 (Fig. 7B). In contrast, a pseudo-bulk comparison of *Neurog2* and *Ppp1r17* aggregated transcript read counts in scRNA-seq data from E10.5 to E17.5 mouse cortices (Di Bella et al., 2021) revealed that, whereas *Neurog2* expression is enriched in cortical NPCs, especially in IPCs, *Ppp1r17* transcripts are for the most part not detected, except for at low levels in IPCs and migrating neurons at early stages (Fig. S1B). Thus, there is a strong correlation between *PPP1R17* and *NEUROG2* transcript levels in human fetal cortices and a weaker correlation in embryonic murine cortices.

### NEUROG2 engages with *PPP1R17* regulatory elements and is sufficient to induce *PPP1R17* transcription

To assess the relationship between *NEUROG2* and *PPP1R17*, we characterized the accessibility of *PPP1R17* upstream regulatory elements by mining single-cell assay for transposase-accessible chromatin using sequencing (ATAC)-seq data generated across cortical cell types in the developing human brain (Ziffra et al., 2021). A comparison to enhancer peaks for *Ppp1r17* in the E12.5 mouse fetal forebrain (Rhodes et al., 2022), and to an ATAC-seq profile of *Ppp1r17* from mouse fetal forebrain at E15.5 (Gorkin et al., 2020) led to the identification of a conserved regulatory region (Fig. 7C, yellow box) upstream of *PPP1R17*, shared open chromatin peaks near the transcriptional start site (TSS; Fig. 7C, green box) and a previously identified HAR that was primarily accessible in glutamatergic neurons and to a lesser extent in IPCs (Fig. 7C). To assess evolutionary constraint, we measured phyloP scores across multiple mammalian species, with negative scores indicative of accelerated evolution for the *PPP1R17*-HAR element in chimps and rhesus monkeys (Fig. 7C), in line with previous analyses of *PPP1R17* (Girskis et al., 2021).

We confirmed that *PPP1R17* was enriched in mCherry-high cells from day 62 COs by qPCR (Fig. 7D). We then performed NEUROG2-ChIP-qPCR on day 45 COs, revealing that NEUROG2 bound both the *PPP1R17*-HAR and the *PPP1R17-*TSS element (Fig. 7E). To test whether this binding was functional, we linked the *PPP1R17*-HAR and -TSS elements to a luciferase reporter (Fig. 7F). Compared to baseline control values, NEUROG2 elevated luciferase activity in SHSY-5Y human neuroblastoma cells using either the *PPP1R17-*HAR or -TSS reporters (Fig. 7F).

To assess whether *Neurog2* was sufficient to induce *PPP1R17* transcription, we transduced day 90 COs with AAV5-GFAP-iCre (control) and AAV5-GFAP-*Neurog2*-T2A-iCre, demonstrating that after 14 days *in vitro PPP1R17* transcript levels increased (Fig. 7G). Notably, similar results were obtained in murine P19 cells (Fig. S1C), suggesting that *Neurog2* is sufficient to transactivate *Ppp1r17* across mammalian species. Finally, to determine whether *NEUROG2* was required to turn on *PPP1R17* in human cortical cells, day 60 COs were transduced with lentiviral constructs carrying a shScr control sequence and two shRNAs targeting endogenous *NEUROG2* (Fig. 7H). After 72 h post-transduction, *PPP1R17* was not significantly affected (Fig. 7H). In summary, *NEUROG2* is sufficient to turn on *PPP1R17* expression, and normally engages with *PPP1R17* regulatory elements in human COs, but other TFs can compensate for the loss of *NEUROG2* to transcribe this HAR-associated gene.

## DISCUSSION

By analyzing transcriptomic data from human fetal cortices and COs, we found that *NEUROG1* and *NEUROG2* are enriched in basal NPCs, similar to findings in the murine cortex (Han et al., 2018). Pseudo-bulk analyses revealed that *NEUROG1* and *NEUROG2* are expressed at roughly equivalent levels in individual cortical NPCs in humans, in contrast to the comparatively higher *Neurog2* transcript levels during murine cortical development. However, the total number of cortical NPCs expressing *NEUROG2* is higher than the number of *NEUROG1*^+^ NPCs in both human (this study) and murine (Han et al., 2018) cortices. Pseudotime trajectory analysis of day 30 (this study) and day 90 (Sivitilli et al., 2020) COs revealed that *NEUROG1* lineages predominate early, whereas *NEUROG2* lineages are enriched later. These findings are in line with the earlier role for *Neurog1* in the developing murine cortex (Han et al., 2018). Whether *NEUROG1* is required to slow down early phases of *NEUROG2*-driven neurogenesis in human cortices via the formation of less efficient heterodimers, as shown in mouse (Han et al., 2018), remains to be determined. These data differ from a previous study performed in human fetal cells, which found that *NEUROG2* and its target genes (e.g. *HES6*, *NEUROD4*, *NHLH1*, *NEUROD1*) are expressed at the highest levels in FACS-enriched cortical bRG (CD15^+^, GLAST^+^, CD133-low) and at negligible levels in IPCs (negative for all three markers) (Johnson et al., 2015). One important difference is that Johnson et al. (2015) relied on cell-surface protein markers to identify and isolate bRG and IPCs, whereas we used transcript-based methods of cell annotation. Furthermore, Johnson et al. (2015) sorted primary human fetal cells, whereas we used a CO model. Regardless of the differences in cell type biases, both studies found that *NEUROG2* is expressed in basal and apical NPCs.

By gene silencing, we showed that *NEUROG2* is required to turn on known neurogenic target genes in COs, and that the same genes could be induced by ectopic *Neurog2* expression. To identify additional *NEUROG2-*regulated genes, we engineered *NEUROG2-mCherry* KI reporter hESCs for CO modeling. We observed a relatively low concordance between mCherry and NEUROG2 protein expression in derivative COs, which declined between day 18 and 45. Nevertheless, *NEUROG2* transcripts were enriched in mCherry-high sorted CO cells, validating the use of this system to trace and isolate *NEUROG2*-lineage cells. Reasons for a lack of complete concordance between *NEUROG2* and mCherry expression could include the tight regulation of *NEUROG2* transcription in 2-3 h oscillatory cycles (Imayoshi et al., 2008), the short-intracellular half-life of NEUROG2 protein (Li et al., 2012), and the negative regulation of NEUROG2 protein translation (Yang et al., 2014). Additionally, slow mCherry chromophore formation and maturation could delay the appearance of mCherry epifluorescence (Hebisch et al., 2013). *NEUROG2-TagRFP* KI induced pluripotent stem cells were used in 2D neural differentiation cultures in a separate study, and the peak overlap between RFP and NEUROG2 protein was observed by day 19, after which RFP expression similarly diminished (Park et al., 2022).

Using a targeted transcriptomic screen, we identified several genes that are differentially expressed in CO cells derived from *NEUROG2*-expressing NPCs, including ECM-associated genes. In rodent cortices, ECM expression levels are high in the VZ, where proliferative, aRG reside, but not in the SVZ, where IPCs have a limited proliferative potential (Arai et al., 2011; Florio et al., 2015; Pollen et al., 2015). In contrast, in gyrencephalic species, bRG and IPCs express high levels of ECM genes to support integrin signaling and to create a pro-proliferative, oSVZ niche (Amin and Borrell, 2020; Arai et al., 2011; Fietz et al., 2012; Martinez-Martinez et al., 2016). As a result, bRG and IPCs, which have lost constraining attachments to the ventricular surface, proliferate extensively to support increased neurogenesis and cortical expansion. We found that although NEUROG2 engages with collagen-gene regulatory elements and is sufficient to induce *COL1A1*, *COL1A2* and *COL3A1* transcription in COs, it is not necessary for their transcription. However, *NEUROG2* silencing increases *COL1A1*, *COL1A2* and *COL3A1* expression, suggestive of a negative regulatory relationship, possibly because silencing *NEUROG2* may have different effects on collagen-gene expression depending on whether the targeted cells are dividing NPCs or post-mitotic neurons, as shown for other NEUROG2 target genes (Péron et al., 2023). Further insights may be gained by assessing the relationship between NEUROG2 and other inducers of ECM gene expression, such as SOX9, which is similarly expressed in basal NPCs in the human and ferret cortex (Guven et al., 2020).

NEUROG2 is expressed in bRG, and human-gained enhancers are enriched in genes expressed in bRG, including Notch signaling genes (Reilly et al., 2015; Song et al., 2020). NEUROG2 and other basic helix-loop-helix TFs, such as ASCL1, control the expression of the Notch ligands *DLL1* and *DLL3* (Castro et al., 2011; Henke et al., 2009), such that these genes were not differentially expressed in our separated mCherry-high and mCherry-low cells. However, this finding does not negate the possibility that these two proneural genes may differentially regulate the expression of Notch ligands in basal NPCs, which could be tested in the future. Our analysis of the top DEGs in mCherry-high COs identified an enrichment of two HAR-associated genes, *PPP1R17* and *FZD8*. Using ChIP-qPCR and overexpression studies, we showed that NEUROG2 binds to the *PPP1R17*-HAR and -TSS, and is sufficient to induce *PPP1R17* expression. *PPP1R17*, a HAR-regulated gene encoding a phosphatase regulatory subunit is expressed in human but not mouse cortical NPCs, at least at the protein level (Girskis et al., 2021). However, based on our pseudo-bulk analyses, *Ppp1r17* transcripts are detected at low level in murine IPCs, and *Neurog2* is sufficient to induce *Ppp1r17* expression in P19 cells. Thus, the correlation between *NEUROG2* and *PPP1R17* in cortical NPCs is not a uniquely human feature.

Taken together, *NEUROG2* has at least some conserved gene targets in mouse cortices and human COs, although further studies will be required to compare target genes across species comprehensively, which may better explain the unique patterns of human neurogenesis.

## MATERIALS AND METHODS

### hESC maintenance

hESCs (H1/WA01) were purchased from WiCell Research Institute, Wisconsin, USA. hESC usage for this project was approved by the Canadian Stem Cell Oversight Committee (SCOC application to C.S. and to C.S. and J.N.) as well as by the SRI's Research Ethics Board (REB Project Identification Number: 5003). Briefly, hESCs were cultured under feeder-free conditions in mTeSR Plus media (Stem Cell Technologies, 100-0276) on plates coated with Matrigel (Corning, 354277) and maintained in 5% CO_2_ incubators at 37°C. Versene (Thermo Fisher Scientific, 15040-066) was used to dissociate hESCs by manual pipetting every 4-5 days for maintenance. hESC cultures were monitored daily for differentiated cells, which were removed by manual scraping. Prior to generating COs, quality control tests were routinely performed on hESC cultures, using a human stem cell pluripotency detection qPCR kit (Sciencecell, 0853) and hPSC genetic analysis kit (Stem Cell Technologies, 07550), according to the manufacturer's protocols.

### CRISPR/Cas9 gene editing

CRISPR genome editing was used to insert an mCherry reporter gene into the 3′UTR of the *NEUROG2* locus. The vector pSpCas9(BB)-2A-GFP (PX458) (Addgene, plasmid #48138), was purchased to target *NEUROG2*. To promote HDR of the Cas9-cleaved *NEUROG2* target locus, we co-electroporated: (1) a CRISPR plasmid containing SpCas9-2A-eGFP and a single guide RNA (sgRNA) to *NEUROG2* that was a fusion of a CRISPR RNA (crRNA) and a trans-activating crRNA (tracrRNA); and (2) a repair template containing homology arms flanking an mCherry reporter cassette. Then, 1.5×10^6^ hESCs were transfected with 1 µg DNA (500 ng Cas9 plasmid and 500 ng linearized donor plasmid) by nucleofection (pulse code CA137) using the P3 Primary Cell 4D-Nucleofector X kit (Lonza, V4XP-3024) in a 4D-Nucleofector (Lonza, AAF-1003B) and were plated in a 6-well plate containing 10 µM Rock inhibitor Y-27632 (Stem Cell Technologies, 72302). Twenty-four hours after transfection, cells were harvested for quantification of transfection efficiency by flow cytometry for EGFP expression, indicative of Cas9 transfection. To isolate individual clones, GFP-positive cells were sorted and re-plated at clonal density in multiple 10-cm plates. Individual clones were then picked and further expanded, followed by genomic DNA extraction to screen for clones that underwent HDR using ddPCR. For ddPCR, we employed a forward primer-probe upstream to the starting point of the homology arm region and a reverse primer-probe that only bound to a site inside the exogenous mCherry sequence. Two correctly targeted clones were used for downstream experiments (lines 105 and 117).

### CO generation

We adapted our CO differentiation protocol according to a previously described protocol (Qian et al., 2018, 2016). For embryoid body (EB) formation on day 0, hESC colonies were dissociated with Gentle Cell Dissociation Reagent (Stem Cell Technologies, 07174) for 7 min at 37°C, and 12,000 hESCs in 100 µl STEMdiff kit EB formation media (Stem Cell Technologies, 08570) supplemented with 50 µM Rock inhibitor Y-27632 (Stem Cell Technologies, 72302) were plated in 96-well V-bottom plates (low-binding) (Greiner Bio-One, 651970). On days 1 and 3, 2 µM dorsomorphin, an inhibitor of BMP type I receptors (ALK2, ALK3, ALK6; ACVR1, BMPR1A, BMPR1B, respectively) (Stem Cell Technologies, 72102) and 2 µM A83-01, an inhibitor of TGFβ type I receptors (ALK4, ALK5, ALK7; ACVR1B, TBFBR1, ACVR1C, respectively) (Stem Cell Technologies, 72022) was added to the media, and cells were cultured for 5 days *in vitro* to induce EB formation. On day 5, single EBs that reached ∼400-600 µm in diameter were selected for neural induction and were transferred to individual wells of 24-well ultra-low attachment plates (Corning, 3473). The media was switched to the STEMdiff kit Induction media containing 1 µM SB431542, an ALK4, ALK5, ALK7 inhibitor (Stem Cell Technologies, 72234) and 1 µM CHIR99021, a GSK3β inhibitor that activates Wnt signaling and limits apoptosis (Delepine et al., 2021; Qian et al., 2016) (Stem Cell Technologies, 72054) and cultured for four more days. On day 9, EBs of ∼500-800 µm diameter that had translucent edges, a sign of neuroepithelial induction, were placed onto a single dimple of an embedding sheet (Stem Cell Technologies, 08579) and 15 µl of Matrigel, an undefined ECM preparation that contains collagens, laminins, other ECM molecules and growth factors (Corning, cat. 354277), was added to encapsulate each CO. Matrigel droplets were incubated at 37°C for 30 min before they were washed into 6-well ultra-low attachment plates (Stem Cell Technologies, 38071) containing STEMdiff kit Expansion media with 1 µM SB431542 and 1 µM CHIR99021. On day 13, individual EBs with clear neuroepithelial cell buds were transferred to each well of a 12-well miniature spinning bioreactor (Qian et al., 2018, 2016) containing STEMdiff kit Maturation media. From day 30, ECM proteins were supplemented in Maturation media by dissolving Matrigel at 1% (v/v) containing human recombinant brain-derived neurotrophic factor (BDNF; PeproTech, AF-450-02). The aggregated cells were referred to as COs from this stage onward and were allowed to develop further in maturation media until the experimental endpoints, as described. Once available, we switched to the STEMdiff Dorsal Forebrain Organoid Differentiation Kit (Stem Cell Technologies, 08620) following the manufacturer's protocol, except that COs were transferred to a 12-well plate with a miniaturized multiwell spinning bioreactor SpinΩ lid after day 14, Matrigel (1% v/v) was added to the media after day 20, and BDNF (20 ng/ml) was added to all media after day 30.

### Cryosectioning and immunostaining

COs were rinsed with ice-cold PBS (without Ca^2+^ and Mg^2+^) (WISENT, 311-010-CL), fixed in 4% paraformaldehyde in PBS (PFA; Electron Microscopy Sciences, 19208) overnight, and immersed in 20% sucrose (Sigma-Aldrich, 84097) in 1× PBS overnight after three washes for 5 min in PBS. COs were embedded in Tissue-Tek Optimal Cutting Temperature (O.C.T.) compound (Sakura Finetek), and 10-μm-thick sections were collected with a Leica CM3050 cryostat (Leica Microsystems Canada Inc.). Samples were collected on Fisherbrand™ Superfrost™ Plus Microscope Slides (Thermo Fisher Scientific, 12-550-15). Cryosections of fixed COs were washed in 0.1% Triton X-100 (Sigma-Aldrich, T8787) in PBS (PBST), then blocked for 1 h at room temperature (RT) in 10% horse serum (WISENT, 065-150) in PBST. Primary antibodies were diluted in blocking solution as follows: SOX2 (1:500, Abcam, ab97959), PAX6 (1:500, BioLegend, 901301), NEUROG2 (1:500, Invitrogen, PA5-78556), DCX (1:500, Abcam, ab18723), COL4 (1:200, Abcam, ab6586), LAM (1:200, Sigma-Aldrich, L9393), mCherry (1:500, SICGEN, AB0040-200) and TUJ1 (1:500, BioLegend, 802001). After 1 h of blocking at RT, slides were incubated with primary antibodies at 4°C overnight. The next day, slides were washed five times for 5 min each wash in PBST, followed by incubation with 1:500 dilutions of species-specific secondary antibodies [Invitrogen: donkey anti-goat IgG, Alexa Fluor 568 (A-11057), donkey anti-rabbit IgG, Alexa Fluor 568 (A-10042) and donkey anti-rabbit IgG, Alexa Fluor 488 (A-21206)] for 1 h at RT. Slides were washed five times in PBST and counterstained with 4′,6-diamidino-2-phenylindole (DAPI; Invitrogen, D1306) and mounted in Aqua-polymount (Polysciences Inc., 18606-20). All images were taken using a Leica DMi8 Inverted Microscope (Leica Microsystems CMS, 11889113).

### Bulk RNA-seq

COs were dissociated into a single-cell suspension using the Worthington Papain System kit (Worthington Biochemical, LK003150) according to the manufacturer's instruction. We collected single-cell suspensions from day 45-47 COs, pooling seven or eight COs per sample. Briefly, prewarmed papain solution with DNase (2.5 ml) was added to the COs in a 60 mm dish. COs were minced with a sterile razor blade into smaller pieces and incubated for 30-45 min at 37°C on an orbital shaker (70 rpm). The tissue suspension was triturated eight to ten times with a P1000 pipette tip to assist the release of single cells. Cell suspensions were transferred to a 15 ml centrifuge tube and an ovomucoid protease inhibitor solution (reagent supplied in the Worthington Papain System kit) was added to stop papain activity. The cells were centrifuged at 300 ***g*** for 7 min and filtered through a 40 µm strainer to remove remaining cell aggregates. Single cells were resuspended in PBS containing FACS buffer (1 mM EDTA, 0.1% bovine serum albumin and Ca^2+^/Mg^2+^-free PBS) with DAPI before flow cytometry analysis. Each sample was sorted into mCherry-positive and mCherry-negative groups. Total RNA was extracted from FACS-isolated cells using the MagMAX-96 total RNA isolation kit (Thermo Fisher Scientific, AM1830). The extracted total RNA was quantified by Qubit 3 Fluorometer with Qubit RNA HS Assay kit. The integrity of total RNA (RIN value) was measured using an Agilent 2100 Bioanalyzer with RNA 6000 Pico kit.

### Targeted transcriptome analysis

Targeted transcriptome sequencing was performed on the Ion S5XL Next Generation Sequencing system with the Ion AmpliSeq Transcriptome Human Gene Expression assay kit (Thermo Fisher Scientific). This assay covers 20,802 human RefSeq genes (>95% of UCSC refGene) with a single amplicon designed per gene target. The gDNA in the RNA sample was digested by ezDNase and the cDNA was synthesized from 10 ng of total RNA using SuperScript IV VILO Master Mix with ezDNase Enzyme kit (Thermo Fisher Scientific). The cDNA libraries were constructed with the Ion Ampliseq Library Kit Plus. The targeted areas were amplified by PCR for 12 cycles. The resulting amplicons were treated with FuPa reagent to partially digest primers. Amplicons were ligated to Ion P1 and IonCode barcode adapters and purified using Agencourt AMPure XP reagent (Beckman Coulter). Barcoded libraries were quantified using the Ion Library TaqMan Quantitation Kit (Thermo Fisher Scientific) and diluted to a final concentration of 80 pM. The sequencing template preparation was done using Ion Chef with Ion 540 Chef Kits. Sequencing was performed for 500 flows on an Ion S5XL Sequencer with Ion 540 chip.

### Next-generation sequencing data analysis of bulk targeted transcriptome data

The Ion Torrent platform-specific pipeline software, Torrent Suite version 5.18.1 (Thermo Fisher Scientific) was used to separate barcoded reads and to filter and remove polyclonal and low-quality reads. Ion Torrent platform-specific plugin, ampliseqRNA (v.5.18.0.0) was used for the alignment of the raw sequencing reads and quantitation of normalized gene expression level (reads per million). DESeq2 was used to analyze differential expression. Principal component analysis, generation of scatter plots and volcano plots, hierarchical clustering and pathway analysis were performed with Transcriptome Analysis Console (TAC) 4.0 software using CHP files.

### FACS and qPCR

To validate our original sort, we collected three pools of 5-day 62 *NEUROG2-mCherry* KI COs, each counted as an independent replicate. Cells were dissociated using papain dissociation kit (Worthington Biochemical, LK003150), following the kit protocol. Briefly, COs were minced and incubated at 37°C with 500 rpm shaking for 30 min, with pipetting to mix every 5 min. Cells were pelleted at 300 ***g*** for 5 min followed by passing through the ovomucoid density gradient and centrifugation at 100 ***g*** for 6 min. The cell pellet was resuspended in HBSS buffer with 0.15% bovine serum albumin and 1 mM EDTA. FACS was performed using BD FACSDiva 8.0.3 software to collect mCherry-positive and -negative cells. RNA was isolated using the QIAGEN RNeasy micro kit (74004) followed by cDNA preparation using SuperScript IV VILO™ Master Mix with ezDNase enzyme, following the kit protocol (Invitrogen, 11766050). qPCR was performed using RT^2^ SYBR green qPCR master mix. The mCherry-specific primers used were: F: 5′-GACTACTTGAAGCTGTCCTTCC-3′; R: 5′-CGCAGCTTCACCTTGTAGAT-3′ (Thermo Fisher Scientific).

### Preparation of snRNA-seq libraries and sequencing

Four batches of five COs were pooled and flash-frozen. Nuclei extraction was performed on frozen tissue as per the manufacturer's instructions (Nuclei Isolation Kit, 10x Genomics). Freshly isolated nuclei were counted using a Countess^®^ II FL Automated Cell Counter (Thermo Fisher Scientific) and immediately processed using the Chromium Next GEM Single Cell 5′ Reagent Kit v2 (10x Genomics, 1000263). For each reaction, 16,500 nuclei were loaded onto GEM Chip K for an expected recovery of 10,000 nuclei. Gel Beads-in-emulsion were generated using the Chromium Controller followed by cDNA generation and amplification (13 cycles) as per the manufacturer's instructions. For each sample, 50 ng of cDNA was used for library generation. Equal molar amounts of each library for all samples were pooled and sequenced at an expected depth of 35,000 reads/nuclei using the Illumina NovaSeq X 10B flow cell system (The Centre for Applied Genomics, The Hospital for Sick Children, Toronto, Canada).

### snRNA-seq data analysis in COs

Seurat v.4.0.1 R package (Han et al., 2021) was used for scRNA-seq analysis. Cells that were of low quality or represented doublets were excluded by filtering out cells with >120,000 and <1000 RNA counts and cells with mitochondrial RNA percentage >15. The samples were integrated using the FindIntegrationAnchors and IntegrateData functions followed by SCTransform. Clustering was performed by the RunPCA, FindNeighbors and FindClusters functions using the first 30 principal components. The 2D projection of the clustering was carried out by the RunUMAP function. Proneural negative, *NEUROG2* or *NEUROG1* single- and double-positive cells were identified with an expression threshold >0. Monocle3 R package was used for a pseudotime analysis using DEGs at an adjusted *P*-value cutoff of 0.001.

### Single-cell pseudo-bulk analysis of murine and human cortical datasets

An scRNA-seq dataset from the developing human cortical brain (Braun et al., 2023) was downloaded from a data repository (https://github.com/linnarsson-lab/developing-human-brain/). An scRNA-seq dataset from the developing mouse cortical brain (Di Bella et al., 2021) was downloaded from Gene Expression Omnibus (GSE153164). In each case, raw counts were CPM-normalized and aggregated by cell type at each time point. The resulting pseudo-bulk dataset (containing CPM-normalized average counts per cell type) was log-transformed for visualizing gene expression patterns across select time points (5-14 weeks post-conception in human, and E10.5 to E17.5 in mouse).

### Analysis of PPP1R17 enhancer peaks

A single-cell ATAC-seq profile of *PPP1R17* across broad cortical cell types in the developing human brain was downloaded from the UCSC Genome Browser (https://genome.ucsc.edu/cgi-bin/hgTracks?db=hg38&lastVirtModeType=default&lastVirtModeExtraState=&virtModeType=default&virtMode=0&nonVirtPosition=&position=chr21%3A15477990%2D16373898&hgsid=2400341615_qfAhtZ2DO1WyK9M3sZt9AQaRAw24; Ziffra et al., 2021). Enhancer peaks for *Ppp1r17* from mouse fetal forebrain at E12.5 were downloaded from the UCSC Genome Browser (https://genome.ucsc.edu/cgi-bin/hgTracks?db=mm10&lastVirtModeType=default&lastVirtModeExtraState=&virtModeType=default&virtMode=0&nonVirtPosition=&position=chr12%3A52713333%2D62634435&hgsid=2400341871_UU4W4mxmItwfPNO7I8aAxgOJYqiu; Rhodes et al., 2022), and an ATAC-seq profile of *Ppp1r17* from mouse fetal forebrain at E15.5 was downloaded from ENCODE 3 (Gorkin et al., 2020) regulation tracks on the UCSC Genome Browser. The conserved regulatory region (Fig. 7C, yellow box) upstream of *PPP1R17* in the human fetal brain was translated to mouse *Ppp1r17* using the LiftOver tool in the UCSC Genome Browser.

### P19 cell transfection

P19 cells were maintained in growth media containing 1× Alpha Modification of Eagle's Medium (AMEM; WISENT, 310-010-CL), 20% fetal bovine serum and 1% penicillin/streptomycin antibiotic. The cells were transfected using Lipofectamine 3000 (Thermo Fisher Scientific, L3000001) with pCIG2 control and pCIG2-*Neurog2* DNA. The cell growth media was changed to fresh media 24 h post-transfection and the cells were harvested 48 h post-transfection.

### RNA isolation, cDNA preparation and qPCR

Total RNA extraction was performed from COs using the QIAGEN RNeasy micro kit (74004), followed by CDNA preparation using the RT^2^ First strand reverse transcription kit (330401). qPCR using specific primers was performed using RT^2^ SYBR green qPCR Kit (Qiagen, 330513). The QIAGEN primers used were: murine *Neurog2* (PPM28944A), *Dll3* (PPM25734G), *Neurod1* (PPM05527D), *Rnd2* (PPM33691A), *Ppp1r17* (PPM28954C), *Col1a1* (PPM03845F), and *Col3a1* (PPM04784B). Human primers were: *NEUROG2* (PPH11564A), *DLL3* (PPH06025A), *DLL1* (PPH06024E), *EOMES* (PPH12647A), *RND2* (PPH05839G), *NEUROD1* (PPH00039E), *COL1A1* (PPH01299F), *COL1A2* (PPH01918B), *COL3A1* (PPH00439F), *PPP1R17* (PPH14658A), *NEUROD4* (PPH16515A), *NEUROG1* (PPH02437A) and *PDGFRA* (PPH00219C). qPCR analysis was performed using 2^−ΔΔCT^ method by normalizing to the CT value of control samples.

### ddPCR

The QX200 Droplet Digital PCR (ddPCR) system (Bio-Rad) was used for all ddPCR reactions. Detailed information for all primers is in Table S5. For HDR screening, the absolute number of *NEUROG2-mCherry* KI gene copies per cell was quantified and normalized to *RPP30* (Bio-Rad, 10031243). Twenty nanograms of genomic DNA was used in a 20 µl PCR reaction containing 900 nM of the forward and reverse *NEUROG2-mCherry* KI and *RPP30* primers, 250 nM of *NEUROG2-mCherry* KI and *RPP30* probes, and 10 µl of 2× ddPCR Supermix for probes (Bio-Rad). Assay mixtures were loaded into a droplet generator cartridge (Bio-Rad), followed by the addition of 70 µl of droplet generation oil for probes (Bio-Rad) into each of the eight oil wells. The cartridge was then placed inside the QX200 droplet generator (Bio-Rad). Generated droplets were transferred to a 96-well PCR plate (Eppendorf), which was heat-sealed with foil and placed in C1000 Touch Thermal Cycler (Bio-Rad). Thermal cycling conditions were as follows: 95°C for 10 min, 44 cycles of 94°C for 30 s, 53°C for 1 min, and 98°C for 10 min. FAM fluorescent signal, which labeled the *NEUROG2-mCherry* KI DNA sequence, and HEX fluorescent signal which labeled the *RPP30* DNA sequence, were counted by a QX200 Droplet Digital reader and analyzed by QuantaSoft analysis software v.1.7.4.0917 (Bio-Rad). Identified positive clones were expanded and underwent further quality checks.

To quantify the absolute number of *mCherry* and *COL1A2* transcripts, RNA from mCherry-high and mCherry-low cell populations were collected from COs, and 10 ng of total RNA was reverse-transcribed in a 10 µl reaction using the SuperScript VILO cDNA Synthesis Kit (Invitrogen). The resulting cDNA was diluted to either 1:5 (*mCherry*) or 1:1500 (*COL1A2*) before amplification. The ddPCR reaction was performed in a 20 µl volume containing 10 µl of 2× QX200 ddPCR EvaGreen Supermix (Bio-Rad), 5 µl of diluted cDNA, and 1 µl each of 4 µM forward and reverse primers and 3 µl of nuclease-free water. Droplet generation was completed as above but with the addition of 70 µl of droplet generation oil for EvaGreen (Bio-Rad). Thermal cycling conditions were as follows: 95°C for 5 min, then 44 cycles of 96°C for 30 s and 56°C (*mCherry*) or 60°C (*COL1A2*) for 1 min, and then 4°C for 5 min, 90°C for 5 min and 4°C for indefinite hold for dye stabilization. EvaGreen fluorescent signal in each droplet were counted and analyzed as described. The copy number of *mCherry* and *COL1A2* transcripts were normalized to the copies per ng of total RNA. All ddPCR analyses were performed at the SRI Genomics Core Facility.

### Luciferase assay

SHSY-5Y human neuroblastoma cells (ATCC, CRL-2266) were plated in 6-well plates and were transfected using Lipofectamine 3000 with 5 µg of pCIG2 *NEUROG2*-expression vectors (Li et al., 2012), luciferase reporter plasmids, 0.25 µg firefly luciferase and 0.125 µg *Renilla* plasmid (transfection control). Two luciferase reporters were designed with *PPP1R17* promoter with and without primate selective element (HAR). A 1.6-kb primate-selective element-containing (4-5.6 kb upstream of human *PPP1R17*) or a 0.7-kb TSS-flanking (−100 to +600) fragment was synthesized and cloned (GenScript Biotech) into the KpnI and SacI sites upstream of the SV40 promoter driving luciferase in pGL3-Promoter (Promega). To generate pCIG2-*NEUROG2* expression vectors, the *NEUROG2* coding-domain sequence (NM_024019) with Kozak consensus was synthesized and cloned (GenScript Biotech) into the SmaI site between the CAG promoter and IRES-EGFP of pCIG2. Cells were harvested at 48 h post-transfection to measure firefly luciferase and *Renilla* activities using the Dual-luciferase Reporter Assay System (Promega, E1910) following the kit instructions, using a TD 20/20 Luminometer (Turner Designs). Firefly luciferase data was normalized to the corresponding *Renilla* values.

### shRNA lentivirus transduction

Day 60 COs were transduced with two shRNA lentiviruses (Origene, SKU TL302977V) to knock down *NEUROG2* and a scrambled shScr control. Briefly, COs were incubated with 1.5×10^5^ TU lentivirus in 50 µl of maintenance media in a 96-well U-bottom plate for 2 h. COs were transferred to a 24-well plate containing 1 ml maintenance media per well and were harvested 72 h post-transduction. RNA was isolated using the QIAGEN RNeasy micro kit (74004), followed by cDNA preparation (RT^2^ first strand kit; Qiagen, 330401) and qPCR using RT^2^ SYBR Green master mix (Qiagen, 330513).

### AAV transduction in COs

Day 90 COs were incubated with 9×10^10^ GC of each AAV in 50 µl of maintenance media in a 96-well U-bottom plate for 1 h. COs were transferred to a 24-well plate with 500 µl maintenance media and were harvested 14 days post-transduction. AAV5-packaged GFAP-*Neurog2*-T2A iCre or GFAP-iCre vectors were used to overexpress *Neurog2* or control, respectively. pAAV-GFAP-*Neurog2*-T2A-iCre was created by replacing the *Neurod1* in pAAV-GFAP-*mNeuroD1*-T2a-iCre (kind gift of Dr Maryam Faiz, Department of Surgery, University of Toronto, Canada) with that of *Neurog2*. The ITR-flanked region included the *GFAP* promoter, *Neurog2* coding-domain sequence, T2A self-cleaving site, iCre sequence, Woodchuck Hepatitis Virus Post-transcriptional Regulatory Element and a bovine growth hormone polyadenylation signal. RNA isolation was carried out using the QIAGEN RNeasy micro kit (74004), followed by cDNA preparation (RT^2^ first strand kit; Qiagen, 330401) and qPCR using RT^2^ SYBR Green master mix (Qiagen, 330513).

### ChIP-qPCR

Day 45 COs were fixed using 2 mM disuccinimidyl glutarate (Sigma-Aldrich, 80424) for 20 min and 1% formaldehyde for 10 min at RT, followed by a 0.125 M glycine quench and three washes in PBS with protease, proteasome and phosphatase inhibitors, including 50 mM sodium fluoride, 0.2 mM sodium orthovanadate, 0.05 mM MG132 (Sigma-Aldrich, M7449), 2 mM PMSF, and 1× Complete protease inhibitor cocktail (Roche, 04 693 116 001). COs were lysed using Tris-EDTA (TE) buffer containing 1% SDS for 20 min at 4°C, followed by sonication with a bioruptor (Diagenode Pico) with 30 s ON/30 s OFF for 20 cycles. Chromatin was centrifuged at 13,000 ***g*** for 10 min and 5% of the supernatant was used as input. Chromatin was precleared using protein G Dynabeads (Invitrogen, 10003D) for 1.5 h and was then incubated with protein G Dynabeads preincubated with 2 µg anti-Neurog2 antibody (R&D Systems, MAB3314) overnight. The beads were washed twice with 0.5 M LiCl wash buffer, twice with 1 M NaCl wash buffer and once with TE buffer. Elution was performed using 1% SDS containing TE buffer at 65°C with 1400 rpm shaking for 15 min. To the eluted sample, we added 11 µl 5 M NaCl and 0.1 µg/µl proteinase K, and then incubated the sample at 42°C for 2 h and overnight at 65°C for reverse crosslinking. A phenol-chloroform extraction was performed, followed by centrifugation at 13,000 ***g*** for 10 min to collect the clear upper aqueous layer. DNA was precipitated by adding 1 µg/µl glycogen, 50 µl 3 M sodium acetate (pH 5.2) and 900 µl isopropanol for 20 min at −20°C followed by centrifugation for 20 min at maximum speed (13,000 ***g***) at 4°C. DNA was washed using 70% ethanol and dissolved in Tris buffer. Qubit quantification was performed and 1 ng/µl was used to perform qPCR using RT^2^ SYBR Green master mix. The ChIP qPCR primers are described in Table S5. ChIP-qPCR fold enrichment was determined using the 2^−ΔΔCT^ method and by normalizing to the negative ORF target as well as to mock (no antibody) controls.

### Tissue clearing and fluorescence microscopy

For imaging of immunolabeled sections, we used a Leica DMI8 fluorescent microscope or a Zeiss Axiovert 200M confocal microscope. For imaging of COs in 3D, we first performed tissue clearing. Briefly, day 42 and 119 COs were fixed in 4% PFA overnight at 4°C. To preserve the tissue protein architecture, samples were cleared using the SHIELD (Stabilization to Harsh conditions via Intramolecular Epoxide Linkages to prevent Degradation) method (Park et al., 2019). Specifically, COs were incubated in SHIELD OFF solution at 4°C with shaking for 24 h. Subsequently, they were incubated in a mixture of SHIELD ON-Buffer and the SHIELD-Epoxy solution (7:1 ratio) at 37°C with shaking for 6 h. Lastly, the samples were incubated in SHIELD ON-Buffer at 37°C with shaking overnight. To carry out tissue delipidation, the samples were passively run down in the Delipidation buffer (LifeCanvas Technologies) for 3 days at RT. Samples were washed in 0.1% PBST three times over 3 h following each incubation and PFA fixation. COs were incubated in primary antibodies diluted in 0.1% PBST at RT for 48 h. Samples were then incubated in conjugated secondary antibodies diluted in 0.1% PBST [Alexa Fluor 488 goat anti-mouse IgG2a (Invitrogen, A-21131), Alexa Fluor 568 donkey anti-rabbit (Invitrogen, A-10042), Alexa Fluor 488 donkey anti-rabbit IgG (H+L) (Invitrogen, A-21206); 1:250] at RT for 48 h. For index matching and to make samples optically transparent, samples were incubated in EasyIndex medium (LifeCanvas Technologies; RI=1.52) at RT overnight. CO images were acquired using an UltraMicroscope Blaze light sheet fluorescence microscope (Miltenyi Biotech) with a 4× objective. The samples were mounted on a small sample stage using photoactivated adhesive (Bondic CNA) and placed into a custom organic imaging medium in the microscope's chamber (Cargille Immersion Liquid; RI=1.52). Two channels were acquired with a 488 nm wavelength and 85 mW power, and 639 nm with 70 mW, for mCherry and SOX2/TUJ1/LAM or COL4, respectively. A 1.67× magnification post-objective lens was employed generating an in-plane resolution of 1.95 μm and a step size of 3.55 μm (scanning protocol parameters: laser sheet thickness=7.1 μm, NA=0.050, and laser width=30% single sided multi-angle excitation).

### Quantification and statistics

Statistical analysis was performed using GraphPad Prism Software version 8.0 (GraphPad Software). For pairwise comparisons, we used unpaired Student's *t*-tests to calculate statistical significance. For multiple comparison between more than two groups, we used one-way ANOVAs with Tukey post-hoc analyses. In all graphs, error bars represent s.e.m. If a *P*-value was less than or equal to 0.05, we considered the result as statistically significant.

### Key resources

A full listing of all reagents and catalog numbers is presented in Table S6.

## Supplementary Material



10.1242/develop.202703_sup1 Supplementary information

Table S2. Identification of differentially-expressed-genes comparing mCherry-low/-high cortical organoid cells, showing log2FC and padj values. These data are from the RNA-seq analysis of mCherry-high and mCherry-low FACS-enriched cells from *NEUROG2-mCherry* KI hESC-derived COs (this study).

Table S3. Gene set enrichment analysis, showing GO Biological Process terms enriched in mCherry_high vs mCherry_low cortical organoid cells. These data are from the RNA-seq analysis of mCherry-high and mCherry-low FACS-enriched cells from *NEUROG2-mCherry* KI hESC-derived COs (this study).

Table S4. Gene set enrichment analysis, showing GO Biological Process terms enriched in mCherry_low vs mCherry_high cortical organoid cells. These data are from the RNA-seq analysis of mCherry-high and mCherry-low FACS-enriched cells from *NEUROG2-mCherry* KI hESC-derived COs (this study).

Table S5. Primers used in this study.

Table S6. Key Resources Table.
