# Supplementary material for: Examining the NEUROG2 lineage and associated gene expression in human cortical organoids
Source: Development. 2025 Jan 16;152(2):dev202703. doi: 10.1242/dev.202703 (PMC11829764; doi:10.1242/dev.202703)
Supplement: Supplementary information [file develop-152-202703-s1.pdf]

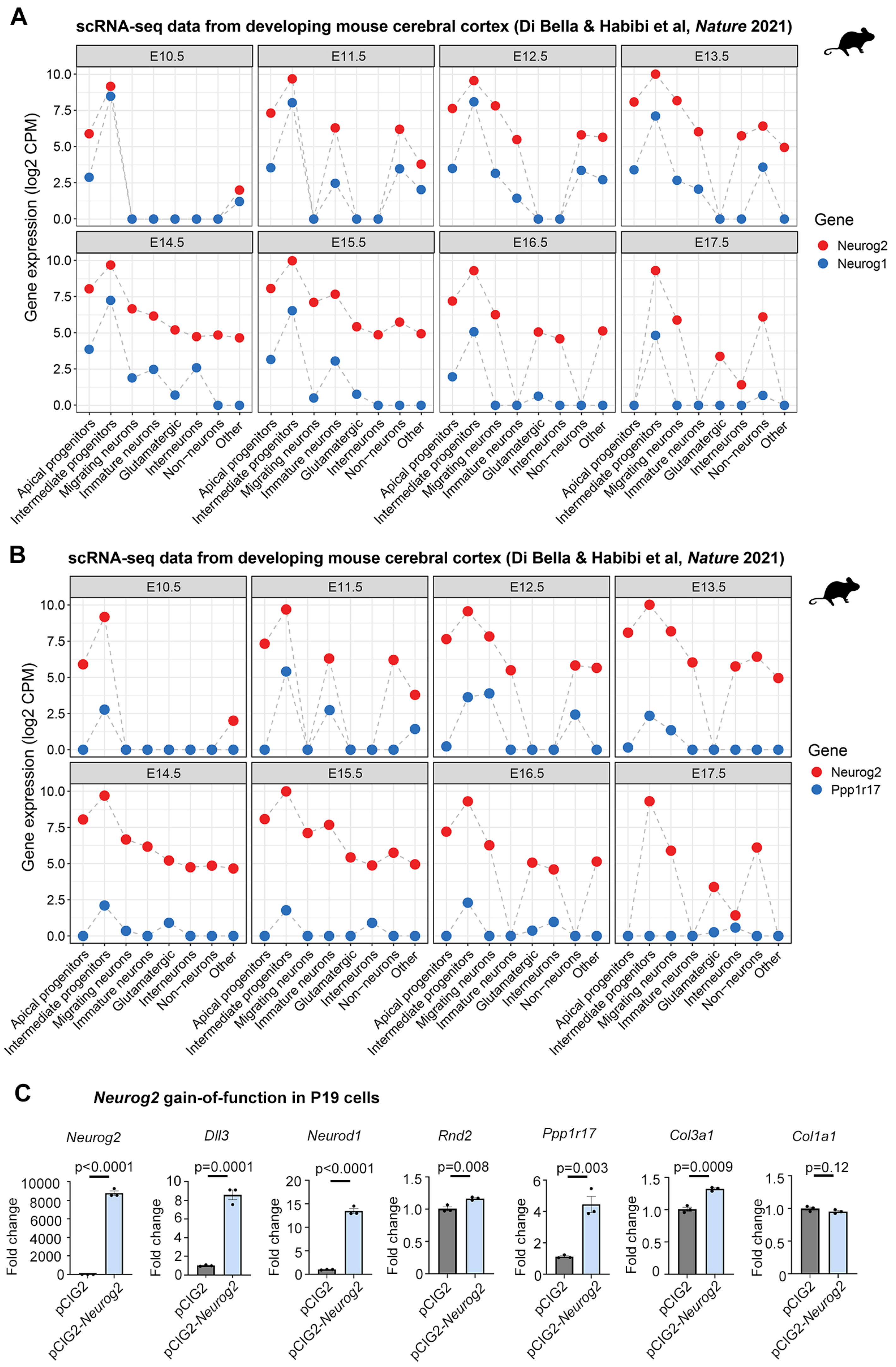

Fig. S1. Comparisons of *Neurog2* expression to *Neurog1* and *Ppp1r17* during mouse cortical development. (**A,B**) Pseudo-bulk analysis of *Neurog2* and *Neurog1* (A) or **Neurog2** and *Ppp1r17* (B) transcript counts in scRNA-seq data collected from E10.5 to E17.5 mouse cortices (Di Bella et al., 2021), showing log2CPM. (C) Graphs showing fold change of *Neurog2* and target genes after overexpression of *Neurog2* in P19 cells (N=3). Graphs show means±SEM. Unpaired student t-tests were used for pairwise comparisons. Significance was defined as p-values less than 0.05.

Development • Supplementary information

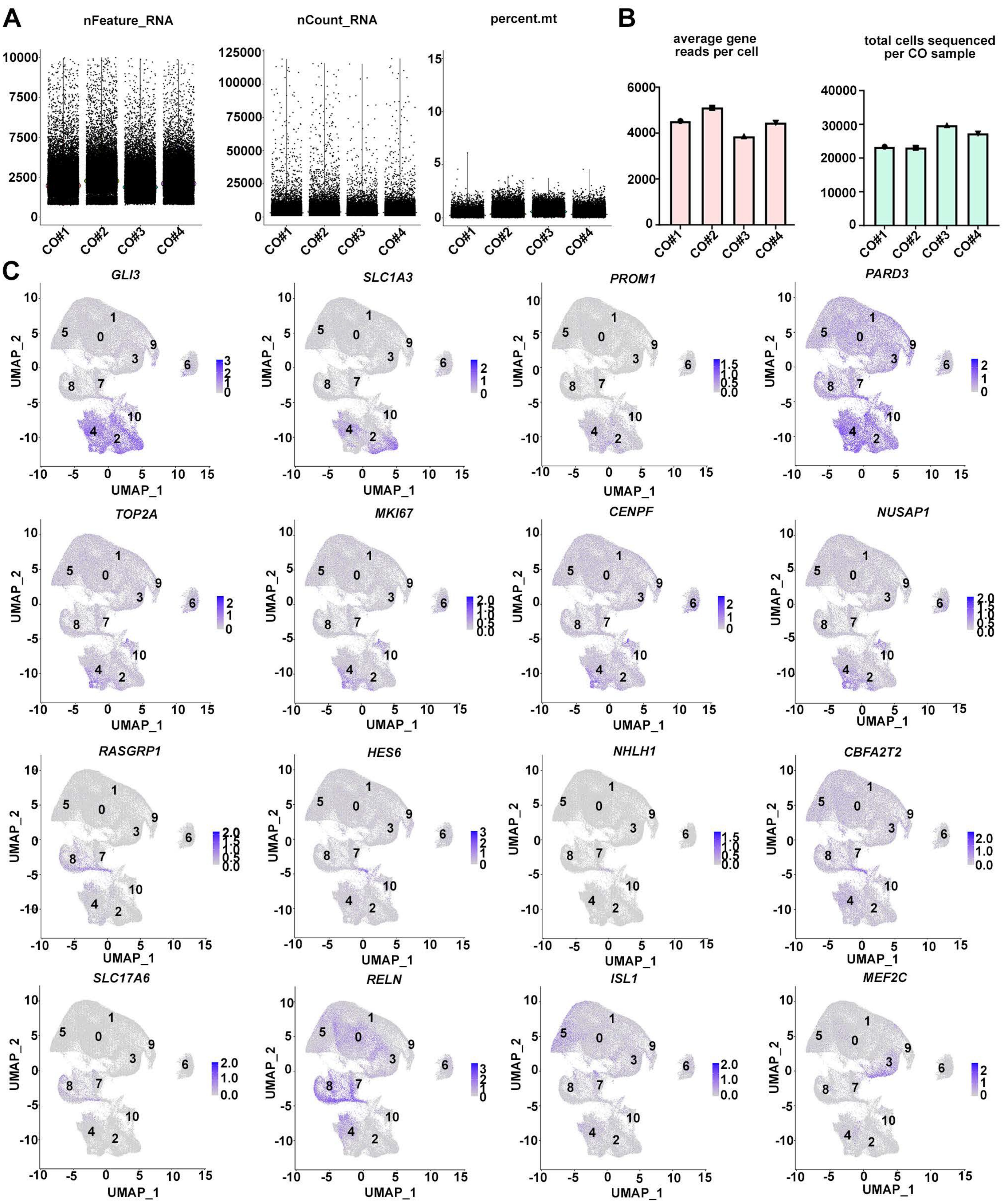

Fig. S2. Analyses of snRNA-seq data collected from day 30 cortical organoids. (**A,B**) Quality control of data collected from the 4 sets of day 30 COs, showing nFeature_RNA (number of genes detected in each sample) and nCount_RNA (total number of UMIs detected within a cell) (A). Average gene reads per cell and total cell sequencing per CO sample for the 4 independent samples are plotted (B). (**C**) Feature plots showing the transcript distributions for aRG-enriched genes (*GLI3*, *SLC1A3*, *PROM1*, *PARD3*), proliferation (*TOP2A*, *MKI67*, *CENPF*, *NUSAP1*), basal NPCs (*RASGRP1*, *HES6*, *NHLH1*, *CBFA2T2*) and neuronal markers (*SLC17A6*, *RELN*, *ISL1*, *MEF2C*).

Development • Supplementary information

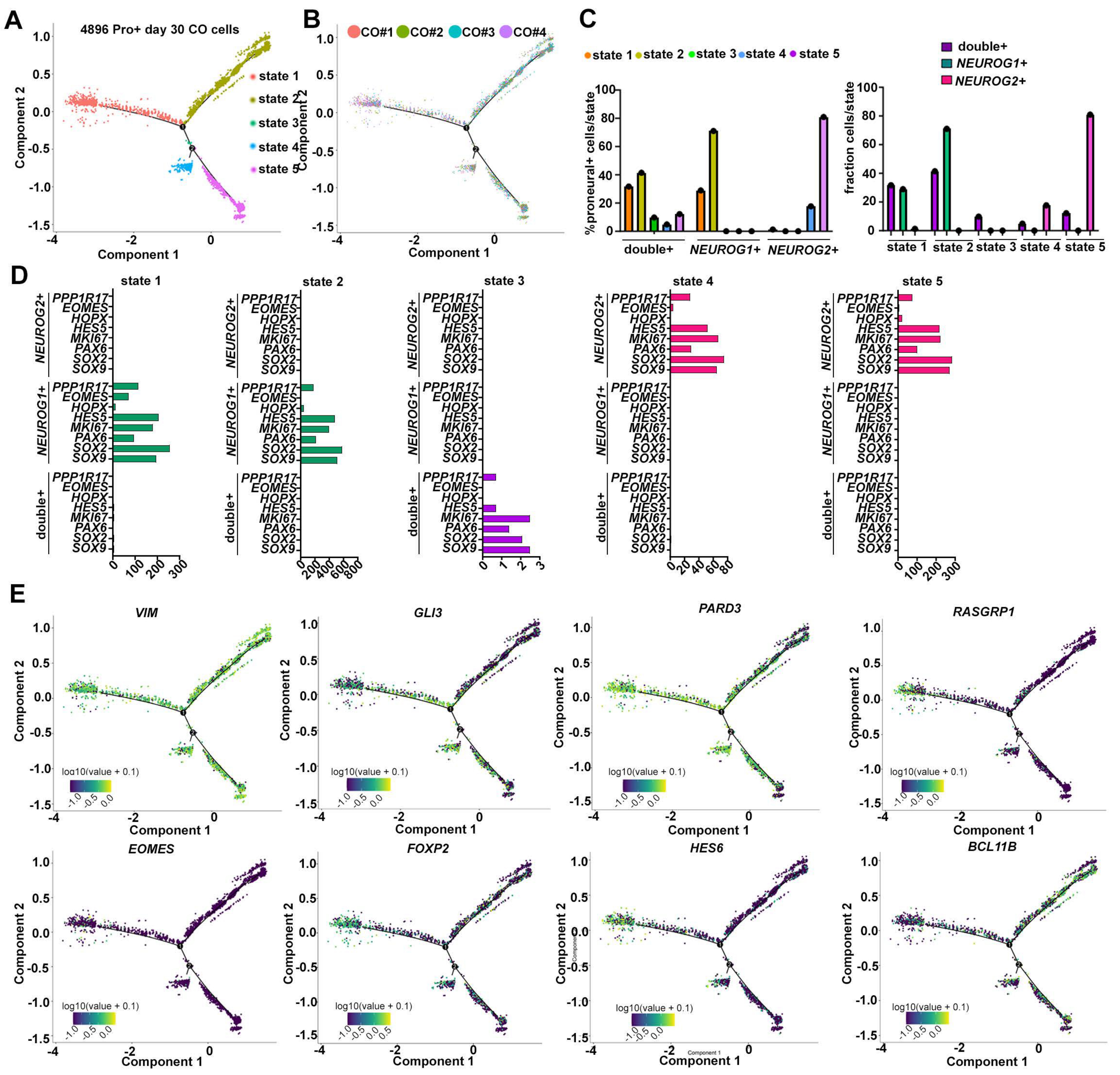

Fig. S3. Pseudotime trajectory analysis of *NEUROG1* and *NEUROG2* expressing cells in day 30 COs. (**A**) Monocle3 lineage trajectory analysis of *NEUROG2*$^{+}$, *NEUROG1*$^{+}$ and double^{+} cells in day 30 CO snRNA-seq data (this study), showing a pseudotime trajectory divided into 5 states, with early (state 1), intermediate (states 2-4) and late (state 5) states. (**B**) Overlay of lineage trajectories for the 4 CO pools shows good overlap. (**C**) The distribution of *NEUROG1* and/or *NEUROG2*-expressing cells in states 1-5. (**D**) Expression levels of other key markers of cortical cell types in each proneural population and cell state. (**E**) Cell type-specific markers were mapped onto the pseudotime trajectory, marking aRG (*VIM*, *GLI3*, *PARD3*), basal NPCs (*RASGRP1*, *EOMES*) and cortical neurons (*FOXP2*, *HES6*, *BCL11B*).

Development • Supplementary information

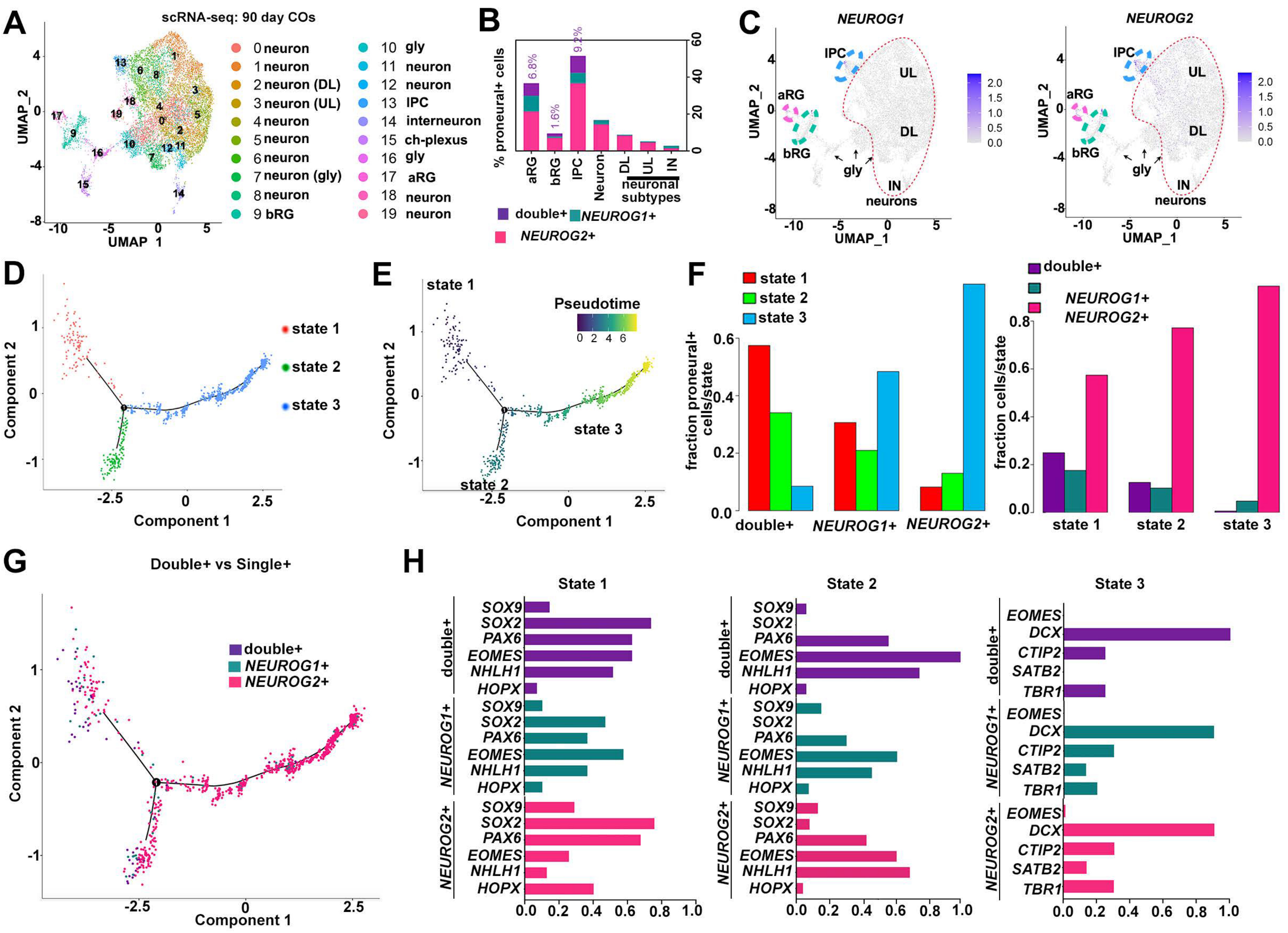

Fig. S4. Pseudotime trajectory analysis of *NEUROG1* and *NEUROG2* expressing cells in day 90 COs. (**A**) UMAP of mined scRNA-seq data collected from 3-month-old cerebral organoids generated with an undirected Lancaster protocol (Sivitilli et al., 2020). Cluster annotations were previously described (Han et al., 2021). (**B,C**) Proportions of annotated cell types in day 90 CO cells expressing *NEUROG1*, *NEUROG2* or both genes (B) Feature plots showing the enrichment of *NEUROG1* and *NEUROG2* transcripts in bRG and IPCs (C). (**D-H**) Monocle3 lineage trajectory analysis of *NEUROG2*$^{+}$, *NEUROG1*$^{+}$ and double^{+} cells in 3-mo cerebral organoid scRNA-seq data (Sivitilli et al., 2020), showing a pseudotime trajectory divided into 3 states (D) with early (state 1), intermediate (state 2) and late (state 3) states (E). The distributions of *NEUROG1* and/or *NEUROG2*-expressing cells in states 1, 2 and 3 are plotted (F) and mapped onto the pseudotime trajectory (G). The expression levels of other key markers of cortical cell types in each proneural population and cell state are plotted (H).

Development • Supplementary information

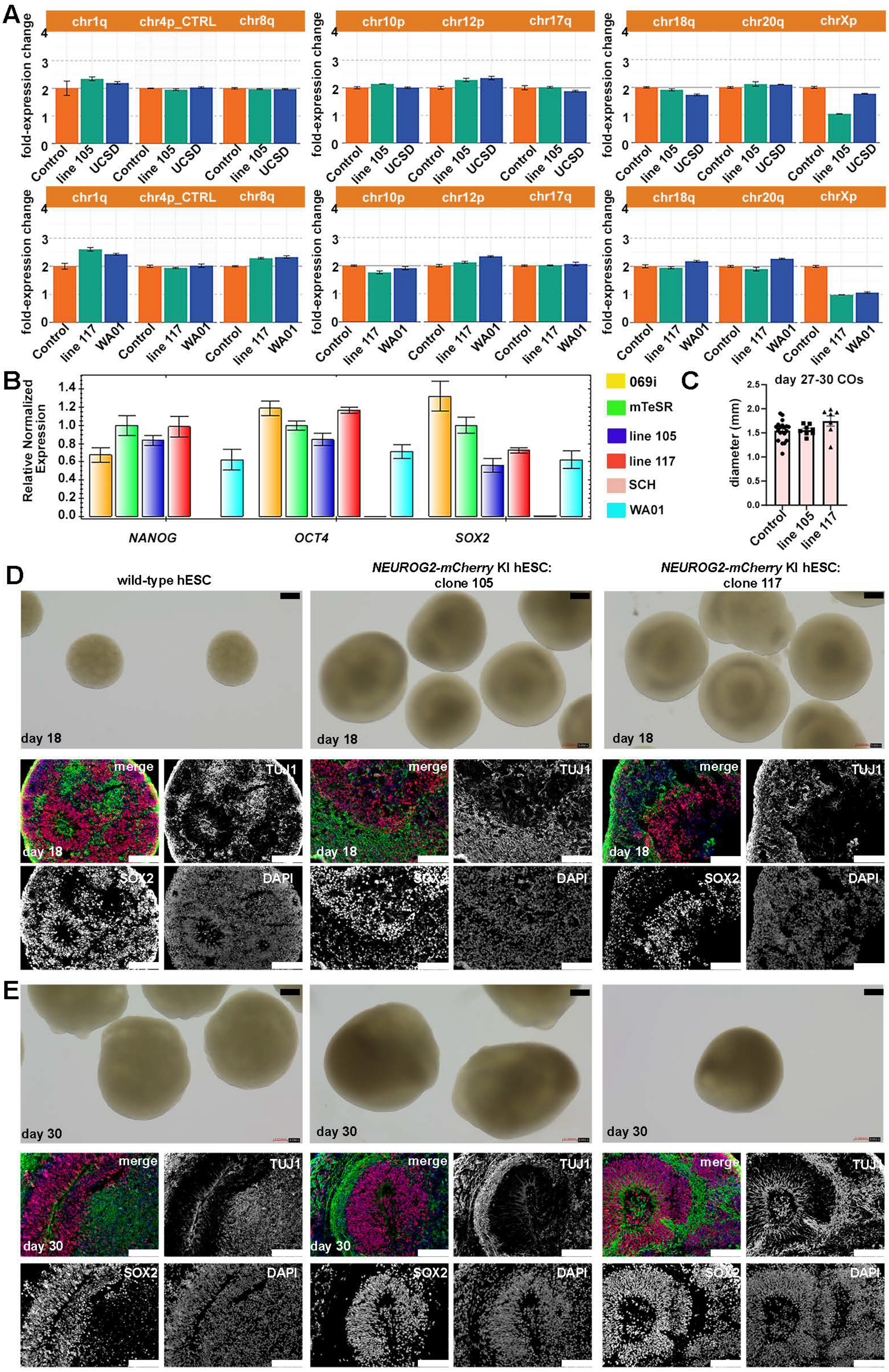
A
chr1q
chr4p_CTRL
chr8q
chr10p
chr12p
chr17q
chr18q
chr20q
chrXp
fold-expression change
Control
line 105
UCSD
line 117
WA01
B
Relative Normalized Expression
NANOG
OCT4
SOX2
069i
mTeSR
line 105
line 117
SCH
WA01
C
day 27-30 COs
diameter (mm)
Control
line 105
line 117
D
wild-type hESC
NEUROG2-mCherry KI hESC: clone 105
NEUROG2-mCherry KI hESC: clone 117
day 18
merge
TUJ1
SOX2
DAPI
E
day 30

Fig. S5. Characterization of *NEUROG2-mCherry* KI hESC cell lines and cortical organoids derived from them. (**A**) *NEUROG2*-mCherry KI hESC cell lines 105 and 117 were validated for the absence of any genomic abnormalities by analysing hotspots for genomic translocations. (**B**) Both lines 105 and 117 expressed pluripotency genes such as *NANOG, OCT4* and *SOX2* at levels similar to the parental WA01 hESC line. Additional controls were UCSD 069i: (UCSD069i-19-3) iPSCs obtained from WiCell, provided by the University of California, San Diego; Schwan cells (SCH) that were used as the negative control and iPSCs maintained in mTeSR Plus media that have passed or checked for good karyotyping and pluripotency. (**C**) Comparison of day 27-30 CO diameters generated from the parental hESC line, or from lines 105 and 117. (**D-E**) Photomicrographs of COs derived from parental hESCs and *NEUROG2-mCherry* KI hESC from line 105 and line 117. Sectioned COs were immunolabeled with SOX2 and TUJ1 antibodies at day 18 (D) and day 30 (E). Scale bars for immunolabelled images -100 µm, Whole mount images -186 µm.

Development • Supplementary information

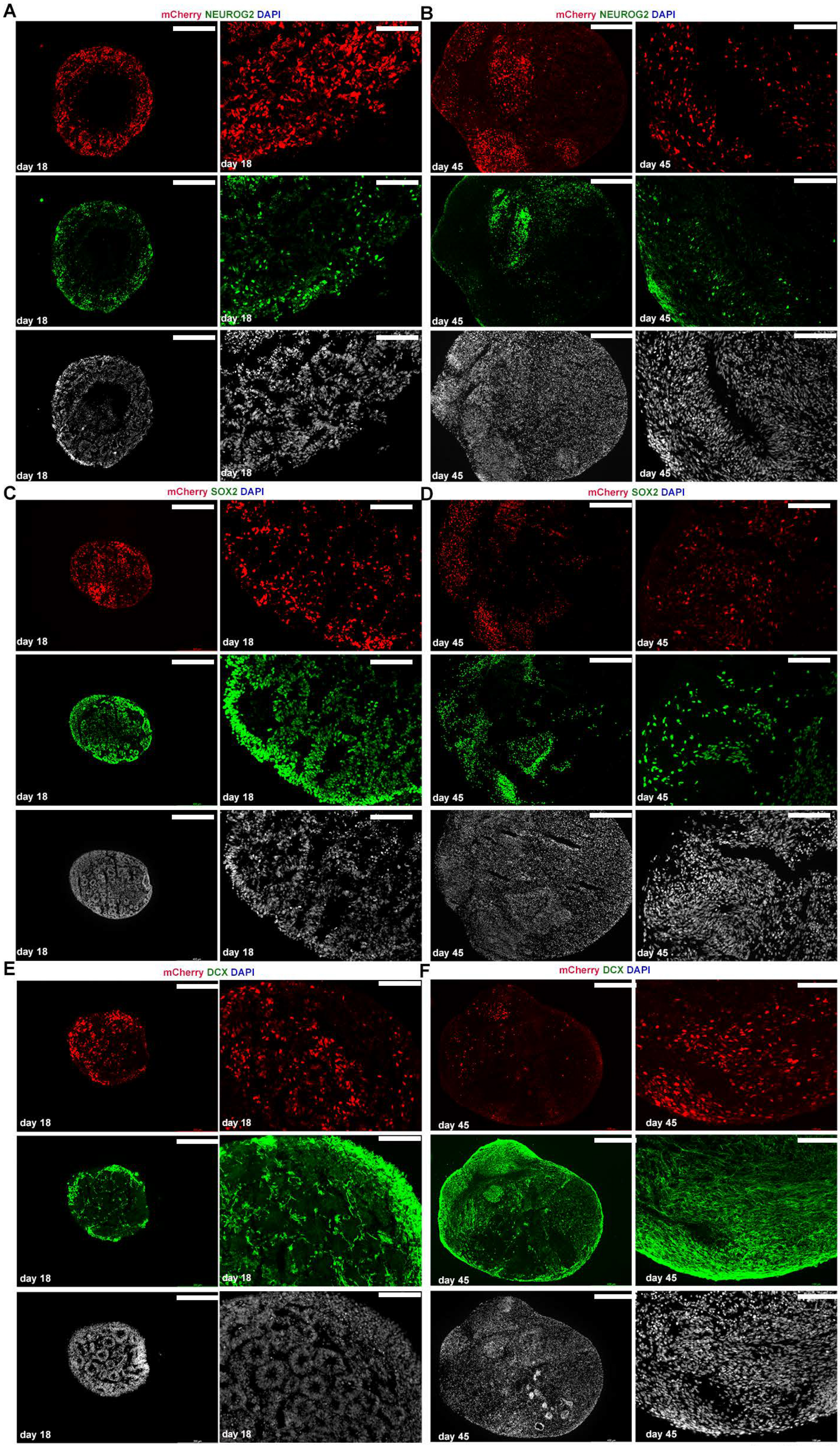

Fig. S6. Characterization of mCherry expression in *NEUROG2-mCherry* KI hESC-derived cortical organoids. (A-F) Co-labeling of day 18 (A,C,E) and day 45 (B,D,F) COs with mCherry and NEUROG2 (A,B), mCherry and SOX2 (C,D) and mCherry and DCX (E,F). Scale bars are 400 µm in low magnification images and 100 µm in high magnification images.

Development • Supplementary information

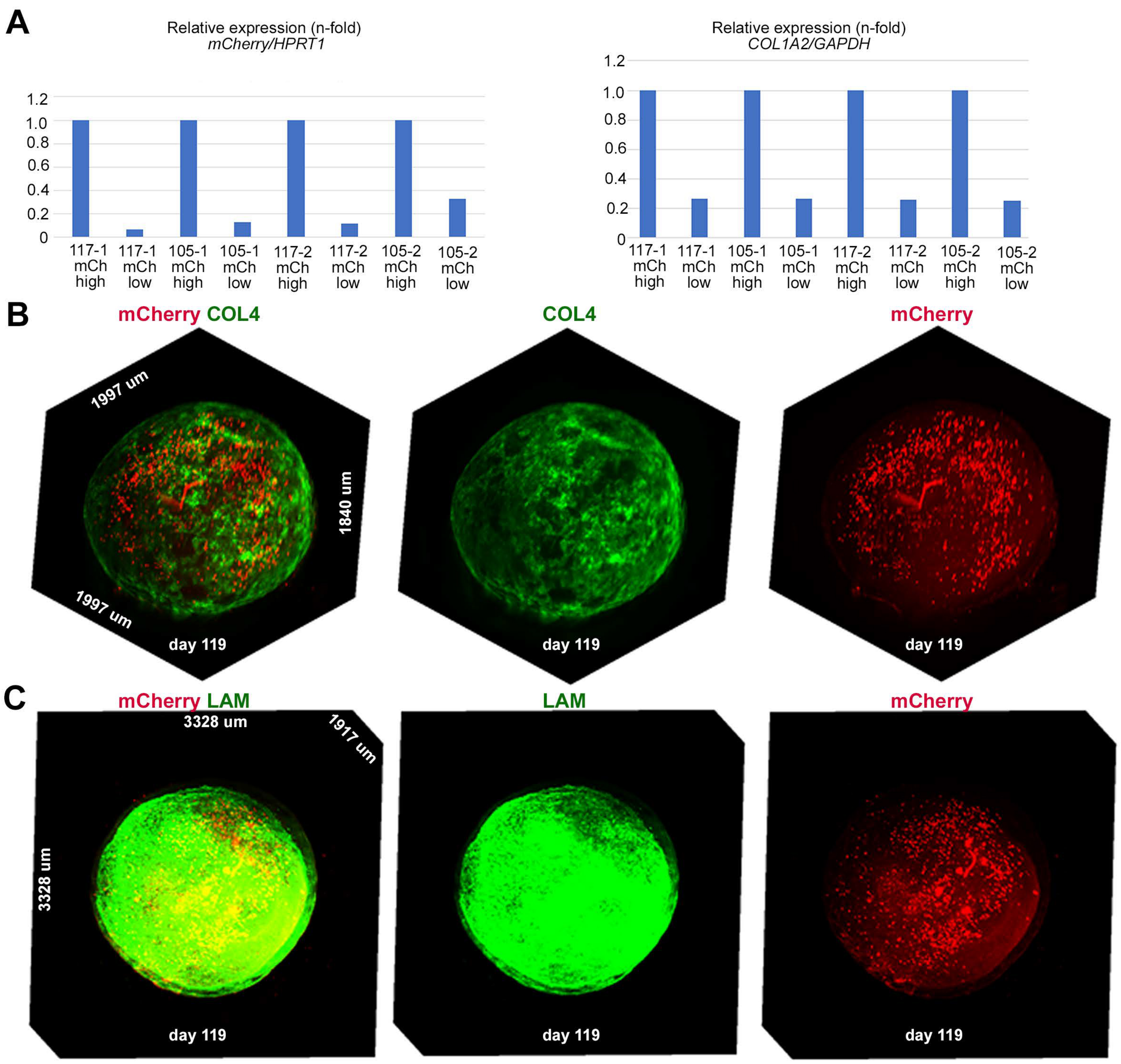

Fig. S7. Expression of ECM markers in *NEUROG2*-mCherry KI hESC derived cortical organoids. (**A**) ddPCR was used to validate FACS-enrichment of *mCherry* transcripts in mCherry (mCh)-high cells collected from two *NEUROG2* mCherry KI hESC cell lines (105 and 117) from different sets of day 45 COs (-1, -2). ddPCR also confirmed the elevated expression of *COL1A2* in mCherry-high versus mCherry-low cells isolated from day 45 COs. (**B,C**) 3D light-sheet imaging of two-clarified day 119 COs immunolabeled for mCherry (red) and COL4 (green, A) or LAM (green, B).

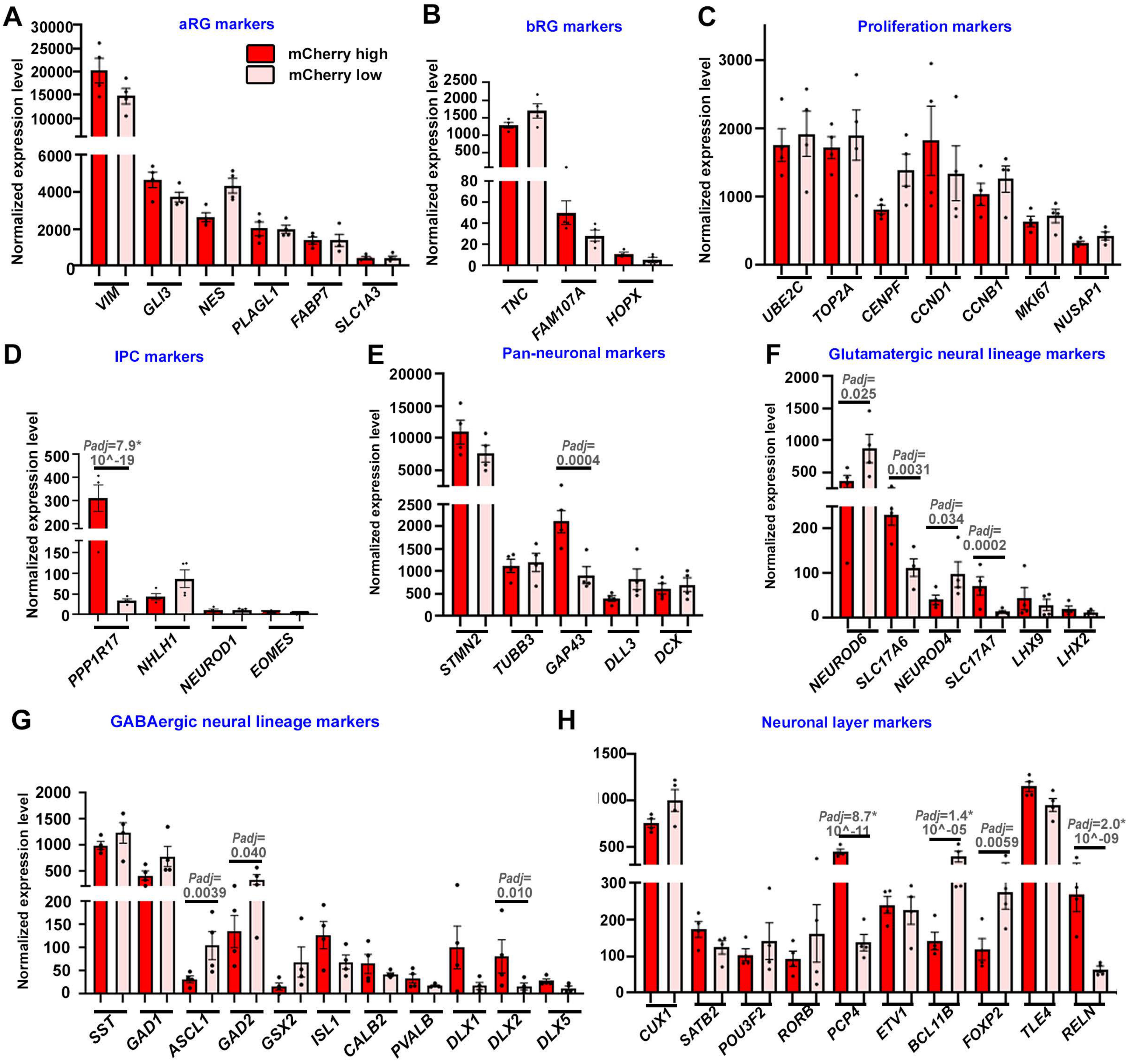

Fig. S8. Comparative analysis of cell type specific markers in mCherry-high cells of *NEUROG2-mCherry* KI hESC derived cerebral organoids. (**A-H**) Bar graphs comparing transcript abundance of cell type specific markers for aRG (A), bRG (B), proliferation (C), IPCs (D), pan-neuronal markers (E), glutamatergic neural lineage markers (F), GABAergic neural lineage markers (G), and neuronal layer markers (H) in *NEUROG2*-mcherry high vs. low cells. Student t-tests were used to individually compare expression levels. p-values: ns - not significant, <0.05 *, <0.01 **, <0.001 ***. Significance was defined as p-values less than 0.05. aRG, apical radial glia; bRG, basal radial glia; IPC, intermediate precursor cells.

Development • Supplementary information

Table S1. List of markers used to assign cortical cell identities in snRNA-seq data from day 30 COs.

CELL TYPE	MARKERS
Forebrain identity	*FOXG1*
Apical radial glia (aRG)	*SOX2, SOX9, PAX6, HES1, VIM, GLI3, NES, PLAGL1, FABP7, SLC1A3, PARD3, PROM1*
Basal radial glia (bRG)	*TNC, FAM107A, HOPX, NHLH1, NEUROD1, CBFA2T2, RASGRP1, TTYH2, PLCB4, SSTR2*
Intermediate progenitor cell (IPC)	*PPP1R17, EOMES*
Dividing NPC	*UBE2C, TOP2A, CENPF, CCND1, CCNB1, MKI67, NUSAP1*
Glutamatergic neuron	*SLC17A6, SLC17A4, TBR1, FOXP2, TLE4, PCP4, ETV1, BCL11B, CUX1, SATB2, POU3F2, RELN, ISL1, MEF2C*
Pan-neuron	*STMN2, TUBB3, GAP43, DLL3, DCX*
Astrocyte	*GFAP, S100b, SLC1A2, GLUL, SOX9, ALDOC*
Oligodendrocyte	*PLP1, PDGFRA, SOX10, MAG, MAL, NFIA, NFIX, NFIC, etc.*
**Extracellular Matrix (ECM)**	*COL1A1, COL1A2, COL28A1, COL3A1, ADAMTSL3, ADAMTS4, MMP15, MMP17, etc.*
**NEUROG2 target genes**	*NEUROD1, NEUROD4, NEUROD6, DLL1, DLL3, RND2, CBFA2T2, HES6, EOMES*

Table S2. Identification of differentially-expressed-genes comparing mCherry-low/-high cortical organoid cells, showing log2FC and padj values. These data are from the RNA-seq analysis of mCherry-high and mCherry-low FACS-enriched cells from *NEUROG2-mCherry* KI hESC-derived COs (this study).

Available for download at
https://journals.biologists.com/dev/article-lookup/doi/10.1242/dev.202703#supplementary-data

Table S3. Gene set enrichment analysis, showing GO Biological Process terms enriched in mCherry_high vs mCherry_low cortical organoid cells. These data are from the RNA-seq analysis of mCherry-high and mCherry-low FACS-enriched cells from *NEUROG2-mCherry* KI hESC-derived COs (this study).

Available for download at
https://journals.biologists.com/dev/article-lookup/doi/10.1242/dev.202703#supplementary-data

Table S4. Gene set enrichment analysis, showing GO Biological Process terms enriched in mCherry_low vs mCherry_high cortical organoid cells. These data are from the RNA-seq analysis of mCherry-high and mCherry-low FACS-enriched cells from *NEUROG2-mCherry* KI hESC-derived COs (this study).

Available for download at
https://journals.biologists.com/dev/article-lookup/doi/10.1242/dev.202703#supplementary-data

Table S5. Primers used in this study.

Available for download at
https://journals.biologists.com/dev/article-lookup/doi/10.1242/dev.202703#supplementary-data

Table S6. Key Resources Table.

Available for download at
https://journals.biologists.com/dev/article-lookup/doi/10.1242/dev.202703#supplementary-data

Development • Supplementary information
