# Supplementary material for: Examining the NEUROG2 lineage and associated gene expression in human cortical organoids
Source: Development. 2025 Jan 16;152(2):dev202703. doi: 10.1242/dev.202703 (PMC11829764; doi:10.1242/dev.202703)
Supplement: Supplementary file 1 [file develop-152-202703_review_history.pdf]

Examining the NEUROG2 lineage and associated gene expression in human cortical organoids

Lakshmy Vasan, Vorapin Chinchalongporn, Fermisk Saleh, Dawn Zinyk, Cao Ke, Hamsini Suresh, Hussein Ghazale, Lauren Belfiore, Yacine Touahri, Ana-Maria Oproescu, Shruti Patel, Matthew Rozak, Yutaka Amemiya, Sisu Han, Alexandra Moffat, Sandra E Black, JoAnne McLaurin, Jamie Near, Arun Seth, Maged Goubran, Orly Reiner, Jesse Gillis, Chao Wang, Satoshi Okawa and Carol Schuurmans
DOI: 10.1242/dev.202703

Editor: Debra Silver

Review timeline

Original submission:	11 January 2024
Editorial decision:	26 February 2024
First revision received:	2 November 2024
Editorial decision:	27 November 2024
Second revision received:	2 December 2024
Accepted:	7 December 2024

Original submission

First decision letter

MS ID#: DEVELOP/2024/202703

MS TITLE: NEUROG2 regulates a human-specific neurodevelopmental gene regulatory program

AUTHORS: Vorapin Chinchalongporn, Lakshmy Vasan, Fermisk Saleh, Dawn Zinyk, Hussein Ghazale, Ana-Maria Oproescu, Shruti Patel, Matthew Rozak, Yutaka Amemiya, Sisu Han, Alexandra Moffat, Sandra E Black, JoAnne McLaurin, Jamie Near, Arun Seth, Maged Goubran, Orly Reiner, Satoshi Okawa, and Carol Schuurmans

I have now received all the referees' reports on the above manuscript, and have reached a decision. The referees' comments are appended below, or you can access them online: please go to BenchPress and click on the 'Manuscripts with Decisions' queue in the Author Area.

As you will see, the referees express considerable interest in your work, but have some significant criticisms and recommend a substantial revision of your manuscript before we can consider publication. If you are able to revise the manuscript along the lines suggested, I will be happy receive a revised version of the manuscript.

All reviewers request mostly minor clarifications and some quantifications/stainings to strengthen conclusions. For example, it is important to further characterize mCherry cells/organoids, as suggested by reviewer 3. Amongst the reviewers, reviewer 3 raises major concerns about the novelty of your findings, including conclusions about differential expression of ECM and mechanisms of Ngn2. Some of this may be addressed with text. However, given these significant concerns, new experiments will be necessary to bolster your conclusions and increase the impact. They do point to specific statements which may help guide your experiments. Along these lines, reviewer 2 also asks you to clarify the mechanisms by which Neurog2 promotes PPP1R17, including knockdown experiments (using human cells and/or luciferase).

© 2024. Published by The Company of Biologists under the terms of the Creative Commons Attribution License (https://creativecommons.org/licenses/by/4.0/).

Your revised paper will be re-reviewed by one or more of the original referees, and acceptance of your manuscript will depend on your addressing satisfactorily the reviewers' major concerns. Please also note that Development will normally permit only one round of major revision. If it would be helpful, you are welcome to contact us to discuss your revision in greater detail. Please send us a point-by-point response indicating your plans for addressing the referees' comments, and we will look over this and provide further guidance.

Please attend to all of the reviewers' comments and ensure that you clearly highlight all changes made in the revised manuscript. Please avoid using 'Tracked changes' in Word files as these are lost in PDF conversion. I should be grateful if you would also provide a point-by-point response detailing how you have dealt with the points raised by the reviewers in the 'Response to Reviewers' box. If you do not agree with any of their criticisms or suggestions please explain clearly why this is so.

Reviewer 1

Advance summary and potential significance to field

In the manuscript “NEUROG2 regulates a human-specific neurodevelopmental gene regulatory program” by Chinchalongporn et al., the authors started to decipher the role of NEUROG2 in human corticogenesis. First, Chinchalongporn et al. provide a solid expression analysis of NEUROG1 and NEUROG2 in human fetal cortices and cerebral organoids and found that NEUROG2 expression is enriched in basal progenitor cells. Then the authors generated a NEUROG2-mCherry Knock-In in human embryonic stem cells for cerebral organoid production. Chinchalongporn et al. used these organoids to FACS isolate cells with high mCherry (high NEUROG2) expression and cells with low mCherry (low NEUROG2) expression to examine the molecular phenotype of cells in the NEUROG2+ lineage. The authors found that mCherry-high cells were enriched for transcripts of the gene PPP1R17 and ECM-related transcripts. Finally, in the mouse model Chinchalongporn et al. could show – while these transcripts are not enriched in mouse Neurog2-mCherry high cells – overexpression of Neurog2 or a mutated (more active) Neurog2 in P19 cells resulted in an induced expression of Ppp1r17 and ECM genes. This suggests that NEUROG2 targets depend on the dosage and activity of NEUROG2. This manuscript clearly provide novel insight into the role of NEUROG2 during human corticogenesis.

Comments for the author

This manuscript presents an interesting experimental approach and is well written. I would consider it suitable for publication in Development. Before publication, I have a few minor points to be considered:

- Throughout the text the authors use the terms aRG (for apical radial glia) and bRG (for basal radial glia).

However, in Figure 1B, the use the alternative terms vRG (for ventral radial glia) and oRG (outer radial glia).

This might be confusing for readers not directly from this field. Could the authors please change it?

- Are the two NEUROG2-mCherry Knock-In human embryonic stem cells lines homozygous or heterozygous for the KI? Do the two lines have the same genotype for the KI (e.g. heterozygous)? Could the authors please add this information?

- The authors claim ?robust co-expression of NEUROG2 and mCherry in neural rosettes? of day 18 cerebral organoids. From the images the authors provided this is difficult to judge and it actually does not look like there is a lot of co-expression. Could the authors please quantify the degree of co-expression to corroborate this claim?

© 2024. Published by The Company of Biologists under the terms of the Creative Commons Attribution License (https://creativecommons.org/licenses/by/4.0/).

- In the same lines, it is difficult to judge the degree of co-expression between mCherry and SOX2 and PAX6 in day 18 cerebral organoids. Could the authors please quantify the degree of co-expression?

- What is the identity of mCherry-positive but SOX2- and PAX6- negative cells in day 45 organoids? Are these basal progenitors and/or neurons? Could the authors please perform co-stainings with basal progenitor and neuronal markers?

- The authors found an enriched expression of PPP1R17 and of some ECM genes in mCherry high cerebral organoid cells. Is it known, if PPP1R17 and the ECM genes are direct targets of NEUROG2? There should be ChIP-Seq or similar data available to check this. This would be important information for the gene regulatory network underlying NEUROG2. This might be also important in the context of the discussion as the authors discuss in detail the target choice of NEUROG2, so it would be important to know if these are indeed direct targets or if this is a secondary effect mediated by other transcription factors.

Reviewer 2

Advance summary and potential significance to field

In this manuscript, the authors aim to dissect the role of NEUROG2 in human cortical neurogenesis. Firstly, they analyzed single cell data from cerebral organoids and human fetal cortex and found that NEUROG2 expression is enriched in basal neural progenitor cells. The authors used a NEUROG2 - mCherry KI human embryonic stem cell to generate cortical brain organoids. They found that PPP1R17 and several ECM genes were elevated in mCherry-high organoid cell. The manuscript contains potentially interesting and important findings, however, there are a few concerns that need to be addressed.

Comments for the author

1) The authors need to show whether NEUROG2 directly promotes PPP1R17 expression by luciferase assay. Alternatively, it is better to show, at least, that the PPP1R17 locus contains the binding sites of NEUROG2.
2) Since PPP1R17 is a HAR-regulated gene, the reviewer did not understand why the authors used P19 cells, which is a mouse cell line, to show the role of Neurog2. The regulatory sequence of Ppp1r17 might be different from that of PPP1R17. Using P19 cells will not address the open question “How gene regulatory networks have evolved to support these human-specific neurodevelopmental features is poorly understood”.
3) Although the reviewer understands that it will take time, preferably, it is better to examine the effect of NEUROG2 knockout/knockdown on PPPR17 expression using human neural stem cells.
4) The authors ignored, or forgot to cite, a previous study in which NEUROG2 was identified as an oRG/bRG-enriched gene (DOI: 10.1038/nn.3980).
5) On page 6, the authors analyzed scRNA-seq data from 90-day old cerebral organoids generated with Lancaster protocol to check NEUORG1 and NEUROG2 expression. However, they used a different brain organoids protocol (Qian et al., 2018) to carry out the experiments. Considering the differences between brain organoid protocols, it would be interesting to know the rationale behind this choice and if the expression pattern of NEUROG1 and NEUROG2 in Qian cortical brain organoids remains similar to Lancaster brain organoids.
6) On page 8, the authors claim that mCherry was co-expressed in the brain organoids neural rosettes referring to Figure 4. However, it would be useful to have separate panels for each IHC marker, to have a better presentation of the co-expression.
7) Figures 5A and B: Is day 18 too early to have a necrotic core? The rosette structures are not clear. It is better to have neural stem cell markers such as Sox2 or Pax6 to visualize the rosettes better.
8) Please elaborate on the conclusion at the end of the first paragraph on page 12 “In conclusion, while mCherry+ cells do not directly co-localize with the ECM proteins, NEUROG2-expressing NPCs transcribe ECM genes that are then translated into proteins that are deposited throughout the cerebral organoid”.

© 2024. Published by The Company of Biologists under the terms of the Creative Commons Attribution License (https://creativecommons.org/licenses/by/4.0/).

9) The authors need to refine references. For example: On page 3, Herculano-Houzel, 2012 does not study cell cycle; on page 4, It is better to cite the original paper (DOI:10.1242/dev.169276) rather than Vaid and Huttner, 2022.
10) A scale bar is missing in Fig. 6A.
11) It is better to show the expression of NEUROG2 in mCherry-high and -low cells as Fig. 5G.
12) What is the rationale for using two different genes (HPRT1 and GAPDH) as an internal control for qPCR analysis?

<u>Reviewer 3</u>

Advance summary and potential significance to field

In this manuscript, the authors have set out to study the role of the pro-neural transcription factor NEUROG2 in human neurodevelopment. Motivated by the observation that the ratio of NEUROG1 and NEUROG2 expression and the duration of expression differ between the mouse and human developing neocortex, the authors generated two knock-in hESC lines that express mCherry from the endogenous NEUROG2 locus to study human development. Based on low and high mCherry expression, transcriptomes were compared. The most prominent gene expression differences relate to the oligodendrocyte lineage program and to ECM expression.
Expression analysis of cell populations isolated from the mouse developing cortex using an analogous strategy confirm inter-species differences in ECM levels. Finally, the authors perform some functional experiments in P19 cells showing that a mutated form of Neurog2, lacking the inhibitory phospho acceptor sites, can induce the expression of one ECM and one intermediate progenitor gene.

The manuscript set out to address an interesting and highly relevant question related to the differential regulation of gene expression programs in basal progenitor cells of the developing neocortex in lissencephalic versus gyrencephalic species. Yet, the presented data largely does not go beyond the prevailing knowledge. It is well established that the pro-neural factor NEUROG2 is expressed in basal progenitor cells in different species. Likewise, the expression differences in ECM between mouse and human are well-known (as cited by the authors). One novel aspect is the characterization of ECM expression in human cerebral organoids for which the authors generate reporter lines. While the lines may potentially present useful tools for the community, they are insufficiently characterized. The title of the study is misleading as the regulation of gene regulatory programs by NEUROG2 is not investigated. Moreover several claims in the study are not sufficiently supported by data.

Comments for the author

The following major points should be considered:

- Figures 1 and 2 mine published RNA-seq data to undermine the well-known fact that Neurog2 is expressed in basal progenitor cells. This can be condensed to two panels in figure 1, focusing on the interspecies differences and expression of NEUROG1 versus NEUROG2.

- The authors may consider to confirm NEUROG1/2 at the protein level, at least for the human organoid system.

- It is unclear whether the human organoids are cerebral (unguided) or cortical (directed to cortical fate by patterning factors)? Instead of referring to Lancaster and Qian protocols, it would be more useful to specify directed which structures are made and according to which protocol (provide reference). If the authors have used a new variation of existing protocols, characterization of the organoids is required with regard to neural identity and cell types.

- The authors have generated two reporter hESC lines with mCherry expression from the endogenous NEUROG2 locus. These new lines should be benchmarked against the parental line and a differentiation time course shown (as in Figure 3).
Immunohistochemistry should be used to confirm that the lines give rise to ventricle-like structures with the expected cell types.

© 2024. Published by The Company of Biologists under the terms of the Creative Commons Attribution License (https://creativecommons.org/licenses/by/4.0/).

- The mCherry-positive cells need to be characterized. This is essential for all further down-stream analysis. What proportion of mCherry-positive cells is SOX2/PAX6, TBR2/NEUROG2 and neural marker positive?

- And the other way around, what proportion of NEUROG2-positive cells is mCherry positive? Is mCherry expressed in all ventricles, or is expression regional within the organoids? Many reporter lines are partially silenced during differentiation to the neural lineage and therefore this information should be transparently provided.

- Do the reporter lines and the organoid culture system recapitulate the NEUROG1/2 expression pattern that is the baseline for the study?

- The difference in mouse versus human ECM expression have been extensively described in the literature (as cited by the authors). What is the novel aspect in this study?

- The RNA-seq data in the mouse neocortex has been produced by similar analogous methods in the past (microdissection, Fietz et al 2012; sorting, Florio et al 2015; reporter lines, Aprea et al, 2013) and in all examples the ECM has already been pointed out. What is the new aspect in this study?

- In the discussion, the authors state that "Using short-term lineage tracing we further found that NEUROG2 regulates a human-specific neurodevelopmental gene regulatory program." There is very little functional data in this manuscript.
The presence of the human expression signatures in the NEUROG2-positive cells does not give any insights into regulation. The induction of two genes upon OE of NEUROG2 in P19 cells does not explain the inter-species difference as Neurog2 is also expressed in the mouse. Any further explanation is speculative and not undermined by data.

- Likewise, the following statement has little support from data: "By demonstrating that NEUROG2 is expressed in IPCs and regulates ECM gene expression, we have found a potential explanation for the expanded ECM production by basal NPCs during human cortical development.". Again, the aspect of differential expression has not been examined.

- "By demonstrating that NEUROG2 is expressed in IPCs and regulates ECM gene expression, we have found a potential explanation for the expanded ECM production by basal NPCs during human cortical development." Which data supports this claim?

- "Taken together, these findings reveal that human NEUROG2 and murine Neurog2 have distinct transcriptional targets and altered functions during neocortical development." Indeed, it would be interesting to know the targets of murine versus human NEUROG2. If the authors want to make that claim, they should provide data on this matter.

- In this context, the authors may wish to include the publication from Noack et al, 2023 on cell type-specific gene regulatory programs in human cortical organoids in the introduction/discussion, which provides insights on the regulation of the epigenetic state by NEUROG2.

- "even though ECM genes are transcribed in germinal compartments in human fetal cortices (Fietz et al., 2012), ECM proteins are deposited in the cortical plate … (Long et al., 2018)." From the three ECM proteins that were studied in Long et al., only one is enriched in the cortical plate, whereas the other two are also present in the germinal zones. This is therefore an incorrect generalization.

Additional points:

- In the introduction, key references on the "multitude of new genes have evolved to drive human brain" are lacking, such as from the Huttner, Kriegstein Pollen, Silver, Vanderhaeghen etc. labs.

- Throughout the paper, different time points are sued for different analysis that are often not matching and sometimes not appropriate to the question. For example, in Figure 1, day 90 Cos

© 2024. Published by The Company of Biologists under the terms of the Creative Commons Attribution License (https://creativecommons.org/licenses/by/4.0/).

appear a bit late to investigate expression in progenitors, such the NPC population are already strongly reduced.

- The labeling of graphs and/or info in legends could be improved. More specific info should be given in the methods (“sufficient cells” is not detailed enough).

- The sequencing data should be deposited in a repository.

- Statistics and replicate info should be check. Three repetitions with the same cell line are not biological replicates.

First revision

Author response to reviewers' comments

Response to Reviewers’ comments:
Reviewer #1:
(Comments for the author): This manuscript presents an interesting experimental approach and is well written. I would consider it suitable for publication in Development. Before publication, I have a few minor points to be considered:
1. “Throughout the text the authors use the terms aRG (for apical radial glia) and bRG (for basal radial glia). However, in Figure 1B, the use the alternative terms vRG (for ventral radial glia) and oRG (outer radial glia). This might be confusing for readers not directly from this field. Could the authors please change it?”
These data have been moved to Suppl. Fig. 4. We have added new snRNA-seq data set collected from day 30 COs in the revised Fig. 2. Throughout these revised figures and in the text, we have used aRG and bRG consistently.
2. “Are the two NEUROG2-mCherry Knock-In human embryonic stem cells lines homozygous or heterozygous for the KI? Do the two lines have the same genotype for the KI (e.g. heterozygous)? Could the authors please add this information?”
Using PCR genotyping, we confirmed that both line 117 and line 105 are heterozygous. Line 117 has correct wild-type and KI amplicon sizes and junction sequences at the 5’ and 3 ends of the mCherry KI (Fig. 4A-D). In line 105, the amplicon sizes are smaller than expected, indicative of a re-arrangement of the targeted 3’UTR region of the NEUROG2 locus. Nevertheless, mCherry was similarly expressed in COs derived from both lines, and PCA revealed that the transcriptomes of mCherry+ cells from these COs were similar (see Fig. 5B).
For these reasons, we only present immunostained data from COs generated from the validated line 117. However, we have used line 105 targeted transcriptome data since the two data sets collected from both lines gave very similar expression patterns (Fig. 5B).
3. “The authors claim robust co-expression of NEUROG2 and mCherry in neural rosettes? of day 18 cerebral organoids. From the images the authors provided this is difficult to judge and it actually does not look like there is a lot of co-expression. Could the authors please quantify the degree of co-expression to corroborate this claim?”
We have now quantified co-expression of NEUROG2 and mCherry in day 18 and day 45 COs (Figure 4F,H) and shown individual channels in Suppl Fig 6. While the co-expression rates are low, this may be due to a delay in mCherry chromophore formation (Hebisch et al., 2013), tightly regulated translation of NEUROG2 protein (Yang et al., 2014), the short, ~30 min half-life of NEUROG2 protein (Li et al., 2012), and/or the 2-3 hr oscillatory cycles of NEUROG2 transcription (Imayoshi et al., 2008). It is likely that these are all contributing factors, as now considered in the Discussion (pg. 20). Nevertheless, we have validated that NEUROG2 transcripts are enriched in mCherry-sorted CO cells, providing confidence that mCherry expression can be used to trace the NEUROG2 lineage (new Fig. 5A).
4. “In the same lines, it is difficult to judge the degree of co-expression between mCherry and SOX2 and PAX6 in day 18 cerebral organoids. Could the authors please quantify the degree of co-expression?”
We have now quantified co-expression of SOX2 and DCX with mCherry in day 18 and day 45 COs (Figure 4F,H) and shown individual channels in Suppl Fig 6.

© 2024. Published by The Company of Biologists under the terms of the Creative Commons Attribution License (https://creativecommons.org/licenses/by/4.0/).

5. “What is the identity of mCherry-positive but SOX2- and PAX6- negative cells in day 45 organoids? Are these basal progenitors and/or neurons? Could the authors please perform co-stainings with basal progenitor and neuronal markers?
We have added DCX counts, as per point 4. Notably, while DCX expression is restricted to newborn neurons in the developing murine cortex, DCX is also expressed in NPCs during ferret cortical development (Wang et al., 2024). We found that in day 18 COs, the majority of mCherry+ cells are both SOX2+ and DCX+, whereas mCherry+ cells are mainly DCX single+ in day 45 COs.
6. “The authors found an enriched expression of PPP1R17 and of some ECM genes in mCherry high cerebral organoid cells. Is it known, if PPP1R17 and the ECM genes are direct targets of NEUROG2? There should be ChIP-Seq or similar data available to check this. This would be important information for the gene regulatory network underlying NEUROG2. This might be also important in the context of the discussion as the authors discuss in detail the target choice of NEUROG2, so it would be important to know if these are indeed direct targets or if this is a secondary effect mediated by other transcription factors.”
We have performed several additional assays to support that PPP1R17 is a direct transcriptional target of NEUROG2.
First, we performed pseudo-bulk comparisons of NEUROG2 and PPP1R17 transcript levels in cortical cells from human fetuses between PCW5 and PCW14 and showed very high overlap (new Figure 7C). This overlap was not observed in mouse cortices examined between E10.5 and E17 (new Suppl Figure 1B).
Second, we performed a ChIP-qPCR experiment and demonstrated that endogenous NEUROG2 binds to known target genes (DLL3, NEUROD4) (new Fig. 3C) and to the PPP1R17-HAR and -TSS in day 45 COs (Fig. 7E).
Third, we cloned both the PPP1R17-HAR and TSS-promoter regions into a luciferase vector. Using this transcriptional reporter system, we demonstrated that NEUROG2 transactivates luciferase reporters containing either the PPP1R17-HAR or -TSS (Fig. 7F).
Fourth, we performed an overexpression study in day 90 COs and showed that Neurog2 induces the expression of PPP1R17 (Fig. 7G). Notably, using shRNA constructs to silence NEUROG2, PPP1R17 was not repressed, suggesting that other genes can compensate for the loss-of NEUROG2 (Fig. 7H).
Taken together, these data demonstrate that PPP1R17 is a bona fide transcriptional target of NEUROG2 in human COs.

Reviewer #2:
1. “The authors need to show whether NEUROG2 directly promotes PPP1R17 expression by luciferase assay. Alternatively, it is better to show, at least, that the PPP1R17 locus contains the binding sites of NEUROG2.”
See response to reviewer #1, comment 6.
2. “Since PPP1R17 is a HAR-regulated gene, the reviewer did not understand why the authors used P19 cells, which is a mouse cell line, to show the role of Neurog2. The regulatory sequence of Ppp1r17 might be different from that of PPP1R17. Using P19 cells will not address the open question “How gene regulatory networks have evolved to support these human-specific neurodevelopmental features is poorly understood”.”
We have repeated the gain-of-function assays using AAV transductions of day 90 COs (new Figure 7A-C). With this system, we found that Neurog2 is sufficient to turn on its known target genes (EOMES, NEUROD1, DLL3, DLL1, RND2) (new Fig. 3E), as well as PPP1R17 (new Fig. 7G). We removed the P19 overexpression data from the manuscript.
3. “Although the reviewer understands that it will take time, preferably, it is better to examine the effect of NEUROG2 knockout/knockdown on PPPR17 expression using human neural stem cells.”
Instead of generating CRISPR-knock-out cells, we used a lentiviral shRNA approach to silence NEUROG2 expression in day 60 COs. We first showed with this system that NEUROG2 is required to turn on known target genes (EOMES, NEUROD1, RND2) (new Fig. 3D). We then used lentiviral gene silencing to show that NEUROG2 is not required to turn on PPP1R17 in human COs, even though it is sufficient, suggestive of compensatory pathways.
4. “The authors ignored, or forgot to cite, a previous study in which NEUROG2 was identified as an oRG/bRG-enriched gene (DOI: 10.1038/nn.3980).”
We apologize for the oversight. We missed this very important paper, which is now prominently discussed in the introduction and discussion sections of the manuscript. We have similarly found that NEUROG2 is expressed in aRG/bRG, as shown by the authors of this study, but also observe an enrichment in IPCs. We discuss differences in the methodologies employed by the two studies that might account for slightly different biases in the expression profiles of NEUROG2 (pg. 20).

© 2024. Published by The Company of Biologists under the terms of the Creative Commons Attribution License (https://creativecommons.org/licenses/by/4.0/).

5. "On page 6, the authors analyzed scRNA-seq data from 90-day old cerebral organoids generated with Lancaster protocol to check NEUORG1 and NEUROG2 expression. However, they used a different brain organoids protocol (Qian et al., 2018) to carry out the experiments. Considering the differences between brain organoid protocols, it would be interesting to know the rationale behind this choice and if the expression pattern of NEUROG1 and NEUROG2 in Qian cortical brain organoids remains similar to Lancaster brain organoids."
We initially used data from day 90 COs generated using the undirected Lancaster protocol (Sivitilli et al., 2020) since Liliana Attisano, the senior author of this study, collaborated with us to use this data and compare NEUROG2 and ASCL1 expression (see (Han et al., 2021)). To directly compare those findings with our current comparison of NEUROG2 and NEUROG1 expression, it was helpful to use the same dataset. However, to analyse the expression of these proneural genes in a model system that more closely resembles human cortical development, we have now conducted a snRNA-seq analysis of day 30 COs generated with a directed differentiation approach for cortical organoids, using a protocol based on the Pasca approach (Birey et al., 2017). Our new snRNA-seq data from day 30 COs is now presented in new Fig. 2.
6. "On page 8, the authors claim that mCherry was co-expressed in the brain organoids neural rosettes referring to Figure 4. However, it would be useful to have separate panels for each IHC marker, to have a better presentation of the co-expression."
We have now shown individual channels for all immunostaining in Suppl. Fig. 6.
7. "Figures 5A and B: Is day 18 too early to have a necrotic core? The rosette structures are not clear. It is better to have neural stem cell markers such as Sox2 or Pax6 to visualize the rosettes better."
Even though we did observe a necrotic core in some day 18 COs, these COs contained SOX2-expressing rosettes, including in COs derived from the parental hESC line (Fig. 2A) or from the NEUROG2-mCherry KI line 117 (Fig. 4E,G; Suppl Fig. 4).
8. "Please elaborate on the conclusion at the end of the first paragraph on page 12 "In conclusion, while mCherry+ cells do not directly co-localize with the ECM proteins, NEUROG2-expressing NPCs transcribe ECM genes that are then translated into proteins that are deposited throughout the cerebral organoid".
We have removed this statement and modified the conclusions of that section.
9. "The authors need to refine references. For example: On page 3, Herculano-Houzel, 2012 does not study cell cycle; on page 4, It is better to cite the original paper (DOI:10.1242/dev.169276) rather than Vaid and Huttner, 2022."
We have gone through and checked the references as indicated. The introduction has been re-written and references validated. The section on cell cycle has been revamped in the introduction on pgs. 3-4.
We have added the older Vaid paper as suggested on pg. 3 "Later, aRG give rise to a secondary pool of intermediate progenitor cells (IPCs) that form a more basally-located subventricular zone (SVZ) (Vaid et al., 2018)."
10. "A scale bar is missing in Fig. 6A."
We thank the reviewer for catching this oversight. However, to refine and refocus the manuscript, we have now removed all mouse data.
11. "It is better to show the expression of NEUROG2 in mCherry-high and -low cells as Fig. 5G."
We now use immunostaining to show NEUROG2 expression in mCherry+ cells in Fig. 5A. AT this level of resolution, we could not detect clear differences in level. Further, to clarify, it was not possible to set an absolute gate for mCherry+ cells using FACS, possibly due to low epifluorescence, so to acknowledge the fact that some mCherry and NEUROG2 transcripts are detected in the "negative" cells, albeit at much lower levels (see new Fig. 5A), we refer to our two sorted populations as mCherry-high and mCherry-low. Our intention was not to suggest that there are variances in mCherry expression within individual NPCs. We have clarified our intent on pgs 12-13.
12. "What is the rationale for using two different genes (HPRT1 and GAPDH) as an internal control for qPCR analysis?"
These ddPCR studies were done by the genomics facility, who select housekeeping genes that have the closest ct values to the target genes. For this reason, HPRT1 was used to normalize mCherry transcripts and GAPDH was used to normalize COL1A2 transcripts. We do not believe that this switch negates the validity of the data, and these analyses remain in Suppl. Fig. 7A. In addition, we have repeated our FACS and demonstrated with a new qPCR analysis that NEUROG2 and mCherry transcripts are enriched in mCherry-high cells, supporting our approach (Fig. 5A).

Reviewer #3:

© 2024. Published by The Company of Biologists under the terms of the Creative Commons Attribution License (https://creativecommons.org/licenses/by/4.0/).

1. “Figures 1 and 2 mine published RNA-seq data to undermine the well-known fact that Neurog2 is expressed in basal progenitor cells. This can be condensed to two panels in figure 1, focusing on the interspecies differences and expression of NEUROG1 versus NEUROG2.”
Firstly, we have expanded our mined data to include day 30 COs, in response to Reviewer 2, comment 5.
We believe that our expanded analysis of single cell transcriptomic data brings several novel and important insights to the field. To our knowledge, a direct comparison of NEUROG1 and NEUROG2 transcript distributions have yet to be reported. Given that these two proneural genes have unique temporal expression profiles and functions during murine cortical development, it is important to begin to understand how they are expressed in a human context (Han et al., 2018). With our comparative approach, using multiple datasets, we can make several novel conclusions about the expression profiles of these two genes: (1) Pseudobulk analyses indicate that NEUROG1 and NEUROG2 are expressed at roughly equivalent levels in individual cortical NPCs in humans, contrasting to the higher expression levels of Neurog2 transcripts during murine cortical development (Fig. 1A, Suppl Fig. 1A); (2) Despite their equivalent expression levels, the total number of cortical NPCs expressing NEUROG2 is higher than NEUROG1+ NPCs, similar to previous findings in the murine cortex (Han et al., 2018), and (3) NEUROG1-expression predominates in early lineages in day 30 COs, whereas NEUROG2 expression predominates in later lineages in day 90 COs. These findings are in line with the temporally-restricted expression profiles and required functions of Neurog1 and Neurog2 in the murine cortex (Han et al., 2018). This information is of importance to researchers who study proneural genes and their dynamic control of neurogenesis.
2. “The authors may consider to confirm NEUROG1/2 at the protein level, at least for the human organoid system.”
We have shown NEUROG2 immunolabeling in Fig. 3A and 4E, G. Unfortunately, we tried several NEUROG1 antibodies and we were unable to observe immunolabeled cells in our COs, likely due to an antibody issue since NEUROG1 transcripts were clearly detected. In any case, after demonstrating that NEUROG2 is the predominant proneural gene during human cortical development in Fig. 1, we focused the remainder of the study on NEUROG2.
3. “It is unclear whether the human organoids are cerebral (unguided) or cortical (directed to cortical fate by patterning factors)? Instead of referring to Lancaster and Qian protocols, it would be more useful to specify directed which structures are made and according to which protocol (provide reference). If the authors have used a new variation of existing protocols, characterization of the organoids is required with regard to neural identity and cell types.”
We have used a dorsal forebrain approach adapted from a protocol published by Sergiu Pasca (Birey F et al. 2017. Assembly of functionally integrated human forebrain spheroids. Nature 545 (7652):54–9). We have added this citation. Furthermore, we generated new snRNA-seq data from the organoids produced with this protocol, validating the expression of FOXG1, a forebrain marker, along with NEUROG1 and NEUROG2, as well as cortical neuronal markers (TBR1, BCL11B). This new data is in Fig. 2 and Suppl. Fig. 2 and 3.
4. “The authors have generated two reporter hESC lines with mCherry expression from the endogenous NEUROG2 locus. These new lines should be benchmarked against the parental line and a differentiation time course shown (as in Figure 3). Immunohistochemistry should be used to confirm that the lines give rise to ventricle-like structures with the expected cell types.”
Supplemental Fig. 5 shows a comparison of COs generated from the parental hESC line and the two NEUROG2-mCherry KI hESC lines.
5. “The mCherry-positive cells need to be characterized. This is essential for all further down-stream analysis. What proportion of mCherry-positive cells is SOX2/PAX6, TBR2/NEUROG2 and neural marker positive?”
See reviewer 1 response #3 and #4 – we have now quantified SOX2, NEUROG2 and DCX in Figure 3.
6. “And the other way around, what proportion of NEUROG2-positive cells is mCherry ? Is mCherry expressed in all ventricles, or is expression regional within the organoids? Many reporter lines are partially silenced during differentiation to the neural lineage and therefore this information should be transparently provided.”
See reviewer 1 response #3 and #4.
7. “Do the reporter lines and the organoid culture system recapitulate the NEUROG1/2 expression pattern that is the baseline for the study?”
We have now carefully quantified NEUROG2 expression using multiple assays and datasets throughout the manuscript, including in mined human RNA-sequencing data. Taken together, our data collected from the COs is consistent with expectations.

© 2024. Published by The Company of Biologists under the terms of the Creative Commons Attribution License (https://creativecommons.org/licenses/by/4.0/).

8. “The difference in mouse versus human ECM expression have been extensively described in the literature (as cited by the authors). What is the novel aspect in this study?” “The RNA-seq data in the mouse neocortex has been produced by similar analogous methods in the past (microdissection, Fietz et al 2012; sorting, Florio et al, 2015; reporter lines, Aprea et al, 2013) and in all examples the ECM has already been pointed out. What is the new aspect in this study?”
We agree with the reviewer that the importance of the ECM gene expression during human cortical development has been elegantly discussed and reported in multiple studies. However, with the exception of a study demonstrating that SOX9 regulates the expression of ECM genes, the transcriptional changes that have allowed for the acquisition of new ECM gene expression in basal NPCs in humans has yet to be shown. The novel aspect of our study is that we have shown that NEUROG2 binds ECM gene promoter regions (COL1A1, COL3A1) in COs, and is sufficient to induce their ectopic expression.
9. “In the discussion, the authors state that “Using short-term lineage tracing, we further found that NEUROG2 regulates a human-specific neurodevelopmental gene regulatory program.” There is very little functional data in this manuscript. The presence of the human expression signatures in the NEUROG2-positive cells does not give any insights into regulation. The induction of two genes upon OE of NEUROG2 in P19 cells does not explain the inter-species difference as Neurog2 is also expressed in the mouse. Any further explanation is speculative and not undermined by data.”
We have now provided functional evidence that NEUROG2 binds the PPP1R17-HAR and that a luciferase reporter construct containing this element is transactivated by NEUROG2. However, we agree that identifying a single human-specific target gene does not warrant a general conclusion about neurodevelopmental gene regulatory programs. We have changed our title to “Identification of new target genes of human NEUROG2 in cortical organoids”.
10. “Likewise, the following statement has little support from data: “By demonstrating that NEUROG2 is expressed in IPCs and regulates ECM gene expression, we have found a potential explanation for the expanded ECM production by basal NPCs during human cortical development.”. Again, the aspect of differential expression has not been examined.”
We have removed this speculation.
11. “By demonstrating that NEUROG2 is expressed in IPCs and regulates ECM gene expression, we have found a potential explanation for the expanded ECM production by basal NPCs during human cortical development.” Which data supports this claim?”
We have removed this statement.
12. “Taken together, these findings reveal that human NEUROG2 and murine Neurog2 have distinct transcriptional targets and altered functions during neocortical development.” Indeed, it would be interesting to know the targets of murine versus human NEUROG2. If the authors want to make that claim, they should provide data on this matter.”
For this study, we have used ChIP-qPCR to validate that NEUROG2 binds to COL1A1, COL3A1 and PPP1R17 regulatory elements but we feel that additional ChIP-seq is beyond the scope of this manuscript.
13. “In this context, the authors may wish to include the publication from Noack et al, 2023 on cell type-specific gene regulatory programs in human cortical organoids in the introduction/discussion, which provides insights on the regulation of the epigenetic state by NEUROG2.”
We have referenced this important article in the introduction.
14. “even though ECM genes are transcribed in germinal compartments in human fetal cortices (Fietz et al., 2012), ECM proteins are deposited in the cortical plate … (Long et al., 2018).” From the three ECM proteins that were studied in Long et al., only one is enriched in the cortical plate, whereas the other two are also present in the germinal zones. This is therefore an incorrect generalization.”
We have removed this statement.

Additional points:
15. “In the introduction, key references on the “multitude of new genes have evolved to drive human brain” are lacking, such as from the Huttner, Kriegstein, Pollen, Silver, Vanderhaeghen etc. labs.”
We have expanded the literature review in the introduction as requested.
16. “Throughout the paper, different time points are sued for different analysis that are often not matching and sometimes not appropriate to the question. For example, in Figure 1, day 90 Cos appear a bit late to investigate expression in progenitors, such the NPC population are already strongly reduced.”

© 2024. Published by The Company of Biologists under the terms of the Creative Commons Attribution License (https://creativecommons.org/licenses/by/4.0/).

We now include single cell transcriptomic data from both day 30 and day 90 COs and show that NEUROG2 expression is higher at the later stage, whereas NEUROG1 predominates early. This data was further validated by qPCR of day 30 and day 90 COs generated using our directed differentiation data.
17. “The labeling of graphs and/or info in legends could be improved. More specific info should be given in the methods (“sufficient cells” is not detailed enough).”
We have edited the methods for clarity. We no longer refer to “sufficient cells”. We ensured that all graph axes are labeled.
18. “The sequencing data should be deposited in a repository.”
We have now deposited the targeted transcriptome and the new snRNA-seq data. Accession numbers are provided.
19. “Statistics and replicate info should be check. Three repetitions with the same cell line are not biological replicates.”
We have now clearly indicated the number of replicates used for each experiment in the Figure legends.

Birey, F., Andersen, J., Makinson, C. D., Islam, S., Wei, W., Huber, N., Fan, H. C., Metzler, K. R. C., Panagiotakos, G., Thom, N., et al. (2017). Assembly of functionally integrated human forebrain spheroids. Nature 545, 54-59.
Borrell, V. and Calegari, F. (2014). Mechanisms of brain evolution: Regulation of neural progenitor cell diversity and cell cycle length. Neuroscience research 86, 14-24.
Calegari, F. and Huttner, W. B. (2003). An inhibition of cyclin-dependent kinases that lengthens, but does not arrest, neuroepithelial cell cycle induces premature neurogenesis. J Cell Sci 116, 4947-4955.
Dehay, C., Giroud, P., Berland, M., Smart, I. and Kennedy, H. (1993). Modulation of the cell cycle contributes to the parcellation of the primate visual cortex. Nature 366, 464-466.
Han, S., Dennis, D. J., Balakrishnan, A., Dixit, R., Britz, O., Zinyk, D., Touahri, Y., Olender, T., Brand, M., Guillemot, F., et al. (2018). A non-canonical role for the proneural gene Neurog1 as a negative regulator of neocortical neurogenesis. Development 145.
Han, S., Okawa, S., Wilkinson, G. A., Ghazale, H., Adnani, L., Dixit, R., Tavares, L., Faisal, I., Brooks, M. J., Cortay, V., et al. (2021). Proneural genes define ground-state rules to regulate neurogenic patterning and cortical folding. Neuron 109, 2847-2863 e2811.
Hebisch, E., Knebel, J., Landsberg, J., Frey, E. and Leisner, M. (2013). High variation of fluorescence protein maturation times in closely related Escherichia coli strains. PLoS One 8, e75991.
Imayoshi, I., Shimogori, T., Ohtsuka, T. and Kageyama, R. (2008). Hes genes and neurogenin regulate non-neural versus neural fate specification in the dorsal telencephalic midline. Development 135, 2531-2541.
Kornack, D. R. and Rakic, P. (1998). Changes in cell-cycle kinetics during the development and evolution of primate neocortex. Proc Natl Acad Sci U S A 95, 1242-1246.
Li, S., Mattar, P., Zinyk, D., Singh, K., Chaturvedi, C. P., Kovach, C., Dixit, R., Kurrasch, D. M., Ma, Y. C., Chan, J. A., et al. (2012). GSK3 temporally regulates neurogenin 2 proneural activity in the neocortex. J Neurosci 32, 7791-7805.
Lukaszewicz, A., Savatier, P., Cortay, V., Giroud, P., Huissoud, C., Berland, M., Kennedy, H. and Dehay, C. (2005). G1 phase regulation, area-specific cell cycle control, and cytoarchitectonics in the primate cortex. Neuron 47, 353-364.
Sivitilli, A. A., Gosio, J. T., Ghoshal, B., Evstratova, A., Trcka, D., Ghiasi, P., Hernandez, J. J., Beaulieu, J. M., Wrana, J. L. and Attisano, L. (2020). Robust production of uniform human cerebral organoids from pluripotent stem cells. Life Sci Alliance 3.
Vaid, S., Camp, J. G., Hersemann, L., Eugster Oegema, C., Heninger, A. K., Winkler, S., Brandl, H., Sarov, M., Treutlein, B., Huttner, W. B., et al. (2018). A novel population of Hopx-dependent basal radial glial cells in the developing mouse neocortex. Development 145.
Wang, W., Yin, C., Wen, S., Liu, Z., Wang, B., Zeng, B., Sun, L., Zhou, X., Zhong, S., Zhang, J., et al. (2024). DCX knockout ferret reveals a neurogenic mechanism in cortical development. bioRxiv, 2024.2006.2013.598795.
Wilson, D. B. and Hendrickx, A. G. (1986). Quantitative aspects of the cell cycle in the cranial neural tube of the rhesus monkey (Macaca mulatta) during early stages of gestation. J Craniofac Genet Dev Biol 6, 363-368.

© 2024. Published by The Company of Biologists under the terms of the Creative Commons Attribution License (https://creativecommons.org/licenses/by/4.0/).

Yang, G., Smibert, C. A., Kaplan, D. R. and Miller, F. D. (2014). An eIF4E1/4E-T complex determines the genesis of neurons from precursors by translationally repressing a proneurogenic transcription program. Neuron 84, 723-739.

Second decision letter

MS ID#: DEVELOP/2024/202703

MS TITLE: Identification of new target genes of human NEUROG2 in cortical organoids

AUTHORS: Lakshmy Vasan, Vorapin Chinchalongporn, Fermisk Saleh, Dawn Zinyk, Cao Ke, Hamsini Suresh, Hussein Ghazale, Lauren Belfiore, Yacine Touahri, Ana-Maria Oproescu, Shruti Patel, Matthew Rozak, Yutaka Amemiya, Sisu Han, Alexandra Moffat, Sandra E Black, JoAnne McLaurin, Jamie Near, Arun Seth, Maged Goubran, Orly Reiner, Jesse Gillis, Chao Wang, Satoshi Okawa, and Carol Schuurmans

I have now received all the referees reports on the above manuscript, and have reached a decision. The referees' comments are appended below, or you can access them online: please go to BenchPress and click on the 'Manuscripts with Decisions' queue in the Author Area.

The overall evaluation is positive and we would like to publish a revised manuscript in Development, provided that the referees' comments can be satisfactorily addressed. Reviewer 2 has remaining concerns about the interpretation of Neurog2-Pppp1r17 experiments, as showing something about "unique" patterns of human neurogenesis (given lack of investigation of the HAR and the mouse expression data which suggest some association). They suggest to remove the HAR results from the manuscript. While this is an option, alternatively I encourage you to consider modifying the emphasis on HARs and modifying some verbiage on human-specific targets, as suggested by the reviewer. Additionally both Reviewers 2 and 3 have just minor suggestions for the manuscript.

Please attend to all of the reviewers' comments in your revised manuscript and detail them in your point-by-point response.If you do not agree with any of their criticisms or suggestions explain clearly why this is so. If it would be helpful, you are welcome to contact us to discuss your revision in greater detail. Please send us a point-by-point response indicating your plans for addressing the referees’ comments, and we will look over this and provide further guidance.

Reviewer 1

Advance summary and potential significance to field

The authors have addressed all my comments and suggestions satisfactorily. I recommend the revised manuscript for publication in Development.

Comments for the author

I do not have any further suggestions.

Reviewer 2

Advance summary and potential significance to field

The authors have addressed many of my previous comments adequately, except for the comment #2.

© 2024. Published by The Company of Biologists under the terms of the Creative Commons Attribution License (https://creativecommons.org/licenses/by/4.0/).

Comments for the author

Since the original version of the manuscript clearly show that “Neurog2 is sufficient to induce Ppp1r17”, the revised manuscript, which contains no description about the results regarding Neurogenin2 in murine cells, is misleading. The authors showed that NEUROG2 binds to both HAR and TSS of PPP1R17, and activates its transcription. No experiments have been performed for the conserved element. Therefore, the contribution of HAR to the expression of PPP1R17 is not clear. In addition, Fig. S1B showed that there is an association between Neurog2 and Pppr17 expression. Taking all the above-mentioned data into consideration, the conclusion would be the following: human NEUROG2 regulates PPPR1R17 expression, and this function is conserved across mammalian species. Even though, the authors claim that NEUROG2 “acquired at least a few novel transcriptional targets that may help to explain its role in driving the unique patterns of human neurogenesis”. This is totally misleading because, in this manuscript, there are no evidence showing that NEUROG2 “acquired” new target genes. In the reviewer’s humble opinion, it is better to remove entire results regarding the HAR from the manuscript, since this is the weakest part of the manuscript. Other parts are sound and contain important information for readers.

Additional comment: the authors must show the exact p value, not just p=0.05, for Fig. 7F HAR graph. If the p value is 0.050, then there is no statistical significance.

<u>Reviewer 3</u>

Advance summary and potential significance to field

This manuscript investigates the role of the pro-neural transcription factor NEUROG2 in human neurodevelopment. In the revised version of the manuscript, the authors have added data to show that NEUROG2 directly binds to some of the target genes, in particular several ECM genes. In addition, they have added additional LOF and GOF experiments and transcriptional reporter assays to strengthen the conclusions on the role of NEUROG2 in regulating ECM genes in human cortical organoids.

Comments for the author

This manuscript has been greatly improved during the revision and most points have been addressed. However, there are still some corrections needed for the citation of the literature.

I had previously requested to include the reference by Noack et al 2023 since it provides insights on the regulation of the epigenetic state by NEUROG2. Even though the authors state in their point-to-point response that they have included the reference in the introduction part, I did not actually find it in the revised manuscript.

“aRG give rise to a secondary pool of intermediate progenitor cells (IPCs)” is followed by a reference to Vaid et al, 2018, which actually studies bRG, not IPCs. Maybe another reference would be more appropriate? Or do the authors generally refer to basal progenitors?

Is the neurogenic window indeed 143 days in humans and all NHPs, given that the length of gestation differs between these species?

“FACS-enriched cortical bRG (CD15+, GLAST+, CD133-low) and at lower levels in IPCs (CD15+, GLAST+,CD133-high) (Johnson et al., 2015).” The CD15+, GLAST+,CD133-high population is actually the aRG population, not the IPCs.
Please correct.

Second revision

<u>Author response to reviewers' comments</u>

© 2024. Published by The Company of Biologists under the terms of the Creative Commons Attribution License (https://creativecommons.org/licenses/by/4.0/).

Reviewer 2 Comments for the author
" Since the original version of the manuscript clearly show that "Neurog2 is sufficient to induce Ppp1r17", the revised manuscript, which contains no description about the results regarding Neurogenin2 in murine cells, is misleading. The authors showed that NEUROG2 binds to both HAR and TSS of PPP1R17, and activates its transcription. No experiments have been performed for the conserved element. Therefore, the contribution of HAR to the expression of PPP1R17 is not clear. In addition, Fig. S1B showed that there is an association between Neurog2 and Pppr17 expression. Taking all the above-mentioned data into consideration, the conclusion would be the following: human NEUROG2 regulates PPPR1R17 expression, and this function is conserved across mammalian species. Even though, the authors claim that NEUROG2 "acquired at least a few novel transcriptional targets that may help to explain its role in driving the unique patterns of human neurogenesis". This is totally misleading because, in this manuscript, there are no evidence showing that NEUROG2 "acquired" new target genes. In the reviewer's humble opinion, it is better to remove entire results regarding the HAR from the manuscript, since this is the weakest part of the manuscript. Other parts are sound and contain important information for readers."
1. We have re-evaluated our data in light of the reviewer's comments, reconsidering data in Figure S1B and data presented in the last iteration of the manuscript, in which we demonstrated that Neurog2 can turn on Ppp1r17 expression in murine P19 cells. We agree that we should alter our conclusion to state that human NEUROG2 regulates PPR1R17 expression, and this function is conserved across mammalian species. We have changed the text to reflect this altered conclusion throughout the manuscript and added back our gain-of-function experiment in murine P19 cells (new Figure S1C). However, we do not think that the HAR data should be removed, as these results are not inconsistent with our new conclusion.

We have edited the manuscript throughout to de-emphasize human-specific targets in the following ways:

- The title was changed to "Examining the NEUROG2-lineage and associated-gene expression in human cortical organoids " instead of "Identification of new target genes of human NEUROG2 in cortical organoids".
- We changed the final sentence in the abstract to: "These data are consistent with a conservation of NEUROG2 function across mammalian species."
- We added the Neurog2 overexpression data in P19 cells back to a new Fig. S1C.
- We added the following to our discussion: "However, based on our pseudobulk analyses, Ppp1r17 transcripts are detected at low level in murine IPCs, and Neurog2 is sufficient to induce Ppp1r17 expression in P19 cells. Thus, the correlation between NEUROG2 and PPP1R17 in cortical NPCs is not a uniquely human feature. "

- We changed the last sentence in the discussion statement to state " Taken together, NEUROG2 has at least some conserved gene targets in mouse cortices and human COs, although further studies will be required to comprehensively compare target genes across species, which may better explain the unique patterns of human neurogenesis.

2. "Additional comment: the authors must show the exact p value, not just p=0.05, for Fig. 7F HAR graph. If the p value is 0.050, then there is no statistical significance."
The rule that we followed is that if a p-value was less than or equal to 0.05, we considered the result as statistically significant. We added this description to our methods section. We have rechecked the p-values for this experiment and the exact p-value is 0.0331, which we have now added to the figure. There was an error in originally transcribing the p-value onto the figure. All other p-values have been rechecked and are accurate.

***** Reviewer 3
1. "I had previously requested to include the reference by Noack et al 2023 since it provides insights on the regulation of the epigenetic state by NEUROG2."
We apologize for this oversight. The Noack paper has now been cited in the introduction as follows: To control fate decisions by cortical NPCs, Neurog1/Neurog2 directly transactivate target genes, and also function as pioneer TFs that open chromatin to facilitate binding by other neurogenic and lineage-specifying TFs (Aydin et al., 2019; Noack et al., 2022; Pereira et al., 2024; Smith et al., 2016). Neurog2 re-organizes the chromatin landscape at several levels, driving DNA demethylation

© 2024. Published by The Company of Biologists under the terms of the Creative Commons Attribution License (https://creativecommons.org/licenses/by/4.0/).

at Neurog2 motifs, especially in distal regulatory regions, and enhancing chromatin looping to increase chromatin accessibility (Manelli et al., 2024; Noack et al., 2022).

2. “aRG give rise to a secondary pool of intermediate progenitor cells (IPCs)” is followed by a reference to Vaid et al, 2018, which actually studies bRG, not IPCs. Maybe another reference would be more appropriate? Or do the authors generally refer to basal progenitors?”
We have corrected the referencing error, citing murine studies, to which this part of the manuscript is referring. We now write “aRG give rise to a secondary pool of intermediate progenitor cells (IPCs) that form a more basally-located subventricular zone (SVZ) and undergo indirect neurogenesis (Haubensak et al., 2004; Kowalczyk et al., 2009; Miyata et al., 2004; Noctor et al., 2004; Noctor et al., 2008).”

3. “Is the neurogenic window indeed 143 days in humans and all NHPs, given that the length of gestation differs between these species?”
What we meant to say was : “To build a larger cortex, the neurogenic window has increased, for example, taking 143 days in human cortices, versus only 11 days in mouse (Ebisuya and Briscoe, 2018; Libé-Philippot and Vanderhaeghen, 2021; Marchetto et al., 2019; Silbereis et al., 2016; Sousa et al., 2017; Toma et al., 2016; Zhu et al., 2018). “ However, to reach the 7000 character limit, this sentence has now been deleted.

4. “FACS-enriched cortical bRG (CD15+, GLAST+, CD133-low) and at lower levels in IPCs (CD15+, GLAST+,CD133-high) (Johnson et al., 2015).” The CD15+, GLAST+,CD133-high population is actually the aRG population, not the IPCs.
Please correct.
We have corrected to “ These data differ from a previous study performed in ferret, which found that NEUROG2 and its target genes (e.g., HES6, NEUROD4, NHLH1, NEUROD1) are expressed at the highest levels in FACS-enriched cortical bRG (CD15+, GLAST+, CD133-low) and at negligible levels in IPCs (negative for all three markers) (Johnson et al., 2015).”

Third decision letter

MS ID#: DEVELOP/2024/202703

MS TITLE: Examining the NEUROG2-lineage and associated-gene expression in human cortical organoids

AUTHORS: Lakshmy Vasan, Vorapin Chinchalongporn, Fermisk Saleh, Dawn Zinyk, Cao Ke, Hamsini Suresh, Hussein Ghazale, Lauren Belfiore, Yacine Touahri, Ana-Maria Oproescu, Shruti Patel, Matthew Rozak, Yutaka Amemiya, Sisu Han, Alexandra Moffat, Sandra E Black, JoAnne McLaurin, Jamie Near, Arun Seth, Maged Goubran, Orly Reiner, Jesse Gillis, Chao Wang, Satoshi Okawa, and Carol Schuurmans

I am happy to tell you that your manuscript has been accepted for publication in Development, pending our standard publication integrity checks.

© 2024. Published by The Company of Biologists under the terms of the Creative Commons Attribution License (https://creativecommons.org/licenses/by/4.0/).
